# GZ7 and GZ8 – Two Zircon Reference Materials for SIMS U‐Pb Geochronology

**DOI:** 10.1111/ggr.12239

**Published:** 2018-10-08

**Authors:** Lutz Nasdala, Fernando Corfu, Blair Schoene, Simon R. Tapster, Corey J. Wall, Mark D. Schmitz, Maria Ovtcharova, Urs Schaltegger, Allen K. Kennedy, Andreas Kronz, Peter W. Reiners, Yue‐Heng Yang, Fu‐Yuan Wu, Sarah E. M. Gain, William L. Griffin, Dawid Szymanowski, Chutimun Chanmuang N., Martin Ende, John W. Valley, Michael J. Spicuzza, Bhuwadol Wanthanachaisaeng, Gerald Giester

**Affiliations:** ^1^ Institut für Mineralogie und Kristallographie Universität Wien Althanstr. 14 Wien A–1090 Austria; ^2^ Department of Geosciences and CEED (Centre for Earth Evolution and Dynamics) University of Oslo P.O. Box 1047 Blindern Oslo 0316 Norway; ^3^ Department of Geosciences Princeton University 219 Guyot Hall Princeton NJ 08544 USA; ^4^ NIGL (Natural Environment Research Council, Isotope Geosciences Laboratory) British Geological Survey Nicker Hill, Keyworth Nottingham NG12 5GG UK; ^5^ Isotope Geology Laboratory Boise State University 1910 University Drive Boise ID 83725 USA; ^6^ Department of Earth Sciences University of Geneva Rue des Maraîchers 13 Geneva CH‐1205 Switzerland; ^7^ John de Laeter Centre Curtin University Building 301, Murdoch Ct. Bentley WA 6845 Australia; ^8^ Geowissenschaftliches Zentrum Georg‐August‐Universität Göttingen Goldschmidtstr. 1 Göttingen D–37077 Germany; ^9^ Department of Geosciences University of Arizona 1040 4th St. Tucson AZ 85721 USA; ^10^ State Key Laboratory of Lithospheric Evolution IGG‐CAS (Institute of Geology and Geophysics, Chinese Academy of Sciences) No. 19, Beitucheng Western Road Chaoyang District Beijing 100029 China; ^11^ CCFS (Australian Research Council Centre of Excellence for Core to Crust Fluid Systems) and GEMOC (Australian Research Council National Key Centre for Geochemical Evolution and Metallogeny of Continents) Department of Earth and Planetary Sciences Faculty of Science and Engineering Macquarie University 12 Wally's Walk Sydney NSW 2109 Australia; ^12^ Department of Earth Sciences Institute of Geochemistry and Petrology ETH Zürich Clausiusstr. 25 Zürich CH–8092 Switzerland; ^13^ Department of Geoscience University of Wisconsin 1215 W. Dayton St. Madison WI 53706 USA; ^14^ Faculty of Gems Burapha University 57 Moo 1, Chon Pratan Rd. Tha Mai Chanthaburi 22170 Thailand; ^15^ Present address: Department of General Science Faculty of Science Srinakharinwirot University 114 Sukhumvit 23 Bangkok 10110 Thailand

**Keywords:** zircon, reference material, SIMS, U‐Pb geochronology, Ti‐in‐zircon geothermometry

## Abstract

Here, we document a detailed characterisation of two zircon gemstones, GZ7 and GZ8. Both stones had the same mass at 19.2 carats (3.84 g) each; both came from placer deposits in the Ratnapura district, Sri Lanka. The U‐Pb data are in both cases concordant within the uncertainties of decay constants and yield weighted mean ^206^Pb/^238^U ages (95% confidence uncertainty) of 530.26 Ma ± 0.05 Ma (GZ7) and 543.92 Ma ± 0.06 Ma (GZ8). Neither GZ7 nor GZ8 have been subjected to any gem enhancement by heating. Structure‐related parameters correspond well with the calculated alpha doses of 1.48 × 10^18^ g^−1^ (GZ7) and 2.53 × 10^18^ g^−1^ (GZ8), respectively, and the (U‐Th)/He ages of 438 Ma ± 3 Ma (2*s*) for GZ7 and 426 Ma ± 9 Ma (2*s*) for GZ8 are typical of unheated zircon from Sri Lanka. The mean U mass fractions are 680 μg g^−1^ (GZ7) and 1305 μg g^−1^ (GZ8). The two zircon samples are proposed as reference materials for SIMS (secondary ion mass spectrometry) U‐Pb geochronology. In addition, GZ7 (Ti mass fractions 25.08 μg g^−1^ ± 0.18 μg g^−1^; 95% confidence uncertainty) may prove useful as reference material for Ti‐in‐zircon temperature estimates.

*In situ* microprobe geochronology by means of SIMS (secondary ion mass spectrometry; Compston *et al*. 1984, Williams 1998) is a comparative method. That is, results of analyses of unknowns need to be calibrated against equivalent analyses of a well‐characterised reference material. Such materials need to be exceptionally homogeneous in isotopic composition, on a scale smaller than the size of SIMS analysis pits (typically comprising ~ 1 ng of material). For zircon (ZrSiO_4_; tetragonal space group *I*4_1_/*amd*) U‐Pb geochronology, however, suitable synthetic reference materials that meet the above requirement are currently not available. To the best of our knowledge, no homogeneous Pb‐doped ZrSiO_4_ crystal has been synthesised thus far. This is explained by the broadly incompatible behaviour of Pb in zircon (Watson *et al*. 1997). Homogeneous Pb‐doped ZrSiO_4_ glass can be synthesised with relative ease, but it is unsuitable as SIMS reference material because the sputtering behaviour of a glass under the $O_{2}^{\text{‐}}$ beam differs appreciably from that of the unknown zircon crystals (Stern and Amelin 2003).

For the above reasons, reference materials for SIMS zircon U‐Th‐Pb geochronology are currently limited to well‐characterised natural zircon. Apart from exceptional isotopic homogeneity, a suitable natural reference material should have a (close to) concordant U‐Pb system and negligible mass fractions of non‐radiogenic Pb (Pidgeon 1997, Kennedy 2000, Kennedy *et al*. 2014, Nasdala *et al*. 2015, Schaltegger *et al*. 2015). Furthermore, the reference material's structural state should be homogeneous and sufficiently similar to that of the unknowns. The latter requirement needs to be checked carefully before a natural zircon sample can be proposed as a new reference material. On the one hand, a SIMS reference material should contain sufficiently high mass fractions of radiogenic Pb. This is advantageous insofar as higher mass fractions result in better counting statistics, which minimise analytical uncertainties and may even allow one to decrease the size of the analysis spots and/or the counting times without losing measurement precision. On the other hand, the emplacement of radiogenic Pb nuclei in the zircon lattice is a destructive process (note that alpha recoils are short‐distance implantations; Weber 1990, Weber *et al*. 1994, Devanathan *et al*. 2006, Valley *et al*. 2015). Therefore, higher mass fractions of radiogenic Pb are typically associated with higher degrees of accumulated self‐irradiation damage, provided no structural reconstitution through thermal annealing has occurred (compare Nasdala *et al*. 2001). Radiation damage will not necessarily affect the material's U‐Th‐Pb isotopic system: even though elevated levels of structural damage enhance the susceptibility of zircon to secondary loss of radiogenic Pb (Krogh and Davis 1975, Nasdala *et al*. 1998, Davis and Krogh 2001, Horie *et al*. 2006), it is well known that radiation damage alone does not cause any Pb loss (note that amorphised but nevertheless concordant zircon has been described by Nasdala *et al*. 2002, 2014, Kostrovitsky *et al*. 2016). However, the potential problem caused by too high levels of radiation damage in the reference material is that significantly different structural states of unknowns and reference material may result in ‘matrix effects’, that is, different ionisation yields and/or U and Pb fractionation during SIMS analysis. Finding a suitable reference material may therefore be a balancing act, as the material should contain enough radiogenic Pb, but still should not have too much radiation damage.

In addition, a suitable SIMS reference material should not have internal fractures, cracks and inclusions, a prerequisite met by highest quality zircon gemstones. SIMS has a comparably low demand of reference material (typically a 100 μm chip will suffice for a 1‐day measurement session); on the other hand, unknowns and the reference material always need to be placed in the same sample mount, which increases the consumption. The intended distribution of multitudes of tiny reference chips to several SIMS laboratories is possible only if a sufficient quantity of material is available. In view of the above, and given the high analytical effort for characterising thoroughly a potential reference material, gemstones to be considered should be sufficiently large. Here, we present measurement results characterising two large zircon gemstones from Sri Lanka, GZ7 and GZ8.

## Samples and preparation

### General description

Zircon samples GZ7 and GZ8 were purchased in 2014 and 2015 from gem traders as cut and faceted gemstone specimens. In our experience, this approach is most expedient. The polished faces of a gem are perfect windows that allow one to examine the specimen's interior, whereas a rough stone cannot be checked in sufficient detail. For reasons elucidated above, our search was focused on large (> 15 ct/> 3 g) gemstones only. Stones to be purchased were first placed in an immersion liquid and carefully inspected with a 10× magnifying lens. Only specimens without visible zoning and seemingly free of inclusions were considered. The shortlisted stones were then subjected to rough mass density measurement and analysis of the degree of broadening of Raman bands. For the semiquantitative evaluation of results that were obtained with a rather basic Raman system in a gem‐testing laboratory in Colombo, whose apparatus function (i.e., instrumental band broadening) was unknown, well‐characterised reference samples were analysed with the same system. Moderately decreased mass densities (*ca*. 4.65–4.50 g cm^−3^) and moderately broadened Raman bands were taken as evidence for significant, but not too high, radiation damage. This, in turn, may indicate the presence of suitably high levels of U and Pb and allows one to exclude thermal gem enhancement.

Based on promising preliminary tests, two gemstones (GZ7 and GZ8) were purchased. Both originated from placer deposits in the Ratnapura district, Sri Lanka (Dahanayake and Ranasinghe 1985, Zoysa 2014). The presumable source area belongs to the Highland Complex (Cooray 1994, Kröner *et al*. 1994, Mathavan and Fernando 2001), which is dominated by Proterozoic rocks that have experienced high‐grade (partially ultrahigh‐temperature) metamorphism during the Pan‐African event at *ca*. 550 Ma (Sajeev *et al*. 2010, Dharmapriya *et al*. 2017, and references therein). The primary source rocks of the gem zircon specimens, however, remain unknown to date.

**Figure 1:**
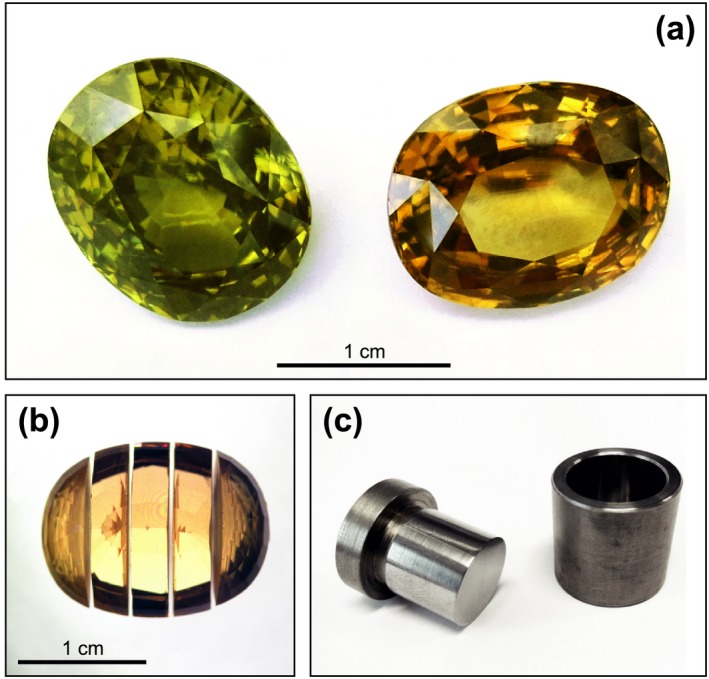
Materials and preparation. (a) Photograph of the two 19.2 ct (3.84 g) zircon gemstones (GZ7: yellow‐brown with orange hue, right; GZ8: yellowish green, left). (b) Image of GZ7 after slicing (photograph A. Wagner). (c) High‐alloy steel cylinder and piston used for mechanical fracturing of slices (piston diameter 18 mm).

The two specimens GZ7 and GZ8 had oval cut, maximum dimensions of 16.8 and 15.9 mm, respectively, and exactly the same mass of 19.2 ct (3.84 g) each (Table 1). Both stones appeared unzoned and flawless, that is, clear and free of inclusions. GZ7 was light brown to dark yellow, with orange hue; GZ8 was yellowish green (Figure 1a). According to the traders, these colours were natural and no heat treatment for colour enhancement had ever been applied.

**Table 1:** General characterisation of zircon specimens GZ7 and GZ8 (Universität Wien) and comparison with well‐crystalline zircon

Parameter	Zircon GZ7		Zircon GZ8		Reference: Ratanakiri^a^	Reference: Synthetic ZrSiO_4_ b
	Natural	Annealed	Natural	Annealed		
Weight (ct mg^−1^)	19.238/3847.6		19.238/3847.6		–	–
Length/width/height (mm)	16.8/13.5/7.8		15.9/13.4/9.4		–	–
Mass density (g cm^−3^)	4.658 ± 0.005		4.537 ± 0.005		4.674 ± 0.005	4.668 ± 0.001
Alpha dose (×10^18^ g^−1^) c	1.48 ± 0.07		2.53 ± 0.11		0.0004 ± 0.0001	–
Unit‐cell dimension *a*_0_ (Å)	6.621 ± 0.002	6.605 ± 0.001	6.673 ± 0.002	6.605 ± 0.001	6.604 ± 0.001	6.606 ± 0.001
Unit‐cell dimension *c*_0_ (Å)	6.025 ± 0.004	5.979 ± 0.001	6.069 ± 0.003	5.982 ± 0.001	5.979 ± 0.001	5.977 ± 0.001
Unit‐cell volume (Å^3^)	264.12 ± 0.22	260.82 ± 0.08	270.26 ± 0.20	260.98 ± 0.07	260.73 ± 0.05	260.82 ± 0.05
Raman shift (cm^−1^) d	1001.8 ± 0.5	1007.9 ± 0.5	996.6 ± 0.5	1007.6 ± 0.5	1008.0 ± 0.5	1008.1 ± 0.5
Raman FWHM (cm^−1^) d	10.9 ± 0.6	2.1 ± 0.3	21.1 ± 1.8	2.1 ± 0.3	1.8 ± 0.2	1.7 ± 0.2
PL (Dy^3+^) FWHM (cm^−1^) d	38 ± 2	12.1 ± 1.0	68 ± 7	11.9 ± 1.0	11.8 ± 0.6	–
PL (Nd^3+^) FWHM (cm^−1^) d	33 ± 2	14.1 ± 1.0	40 ± 3	14.4 ± 1.0	14.3 ± 1.4	–

Quoted uncertainties are 2*s*.

a Mass density, alpha dose and unit‐cell parameters for the well‐crystallised Ratanakiri, Cambodia, zircon are from Zeug *et al*. (2018); PL FWHM values are from Lenz and Nasdala (2015).

b Unit‐cell parameters for pure, undoped ZrSiO_4_ are from van Westrenen *et al*. (2004). Unit‐cell parameters were converted to a theoretical ‘X‐ray density’, which is quoted as reference value for the mass density.

c Calculated according to Murakami *et al*. (1991) from the present U and Th mass fractions and the U‐Pb age.

d Raman spectral parameters are quoted for the ν_3_(SiO_4_) Raman band. FWHM = full width at half‐maximum. PL data are quoted for the ~ 17210 cm^−1^ sublevel of the ^4^F_9/2_ → ^4^H_13/2_ emission of Dy^3+^ and the ~ 11360 cm^−1^ sublevel of the ^4^F_3/2_ → ^4^I_9/2_ emission of Nd^3+^, respectively.

### Sample preparation

The two stones were cut into ~ 2.8‐mm‐thick slices using a Struers AWS1 abrasive wire saw, with a 0.17‐mm‐diameter high‐grade steel wire coated with 20 μm diamond grains (Well Diamantdrahtsägen GmbH, Mannheim). The slicing was done perpendicular to the longest dimension of the gemstone (Figure 1b) to minimise mass loss, that is, the crystallographic orientation was not considered. After washing in pure ethanol and ultrasonic cleaning in distilled water, some slices were loaded into a high‐alloy steel cylinder and piston (steel type 1.2842 90MnCrV 8; Figure 1c) and fractured by gently applying pressure in a mechanical squeezing machine. One large slice per sample was polished on one side, for EPMA (electron probe microanalysis), LA‐ICP‐MS (laser ablation‐inductively coupled plasma‐mass spectrometry) and spectroscopic measurements. For this, the slices were attached to a round one‐inch glass slide using acetone‐soluble glue, surrounded by an acrylic glass ring of corresponding thickness. Fine grinding of these two prepared slices was done using diamond‐coated stainless steel discs, and polishing was done with 3 and 1 μm diamond powder on cloth (whereas polishing on non‐ferrous metal discs could introduce minor amounts of Pb to the materials). After finishing the analyses, slices were detached from their glass slides and, after removal of remnant carbon by mechanical polishing, were subjected to ethanol washing and ultrasonic cleaning.

To estimate the degree of radiation damage and associated parameter changes, small chips of GZ7 and GZ8 were subjected to dry heating in air at 1400 °C for 96 h for structural reconstitution. Samples were placed in a Pt crucible (note that annealing zircon in an alumina crucible may, as an analytical artefact, result in surficial decomposition into oxides; Váczi *et al*. 2009) and heated at a rate of 30 °C min^−1^ to the designated temperature. After 4 days, the furnace was shut off and samples cooled slowly. Slow heating and cooling were preferred to avoid the possible build‐up of strain during shock heating or quenching.

## Analytical techniques

### Electron probe microanalysis imaging and major‐element analysis

A JEOL 8900 RL EPMA (Universität Göttingen) was used for obtaining BSE (back‐scattered electrons) and CL (cathodoluminescence) images and for determining mass fractions of major elements by means of wavelength‐dispersive X‐ray analysis. Imaging was done by scanning the fully focused beam at 20 kV accelerating voltage and 50 nA beam current. For point analyses, the system was operated at 20 kV accelerating voltage and 80 nA beam current, with the electron beam focused to a 10 μm spot. For each sample, 84 point analyses were done along two 13 and 8 mm traverses (that were oriented perpendicularly to each other) across the large polished slice. The measured element‐specific lines (with synthetic or natural calibrant materials and peak/background counting times quoted in brackets) included Al‐Kα (Al_2_O_3_; 120 s; 120 s), Si‐Kα (ZrSiO_4_; 15 s; 10 s), P‐Kα (ScPO_4_; 300 s; 300 s), Ca‐Kα (wollastonite; 120 s; 120 s), Fe‐Kα (haematite; 240 s; 240 s), Y‐Lα (YAG, yttrium aluminium garnet; 300 s; 300 s), Zr‐Lα (ZrSiO_4_; 30 s; 30 s), Yb‐Lα (YbPO_4_; 300 s; 300 s), Hf‐Mα (HfSiO_4_; 60 s; 60 s), Th‐Mα (ThSiO_4_; 300 s; 300 s) and U‐Mα (UO_2_; 300 s; 300 s). Data were reduced using the CITZAF routine in the JEOL software, which is based on the Φ(ρZ) method (Armstrong 1991, 1995). For element mapping (660 × 450 analyses of GZ7, 650 × 600 analyses of GZ8), the system was operated at 20 kV and 300 nA. The beam diameter was 10 μm, the step width was 20 μm, and the dwell time was 100 ms.

### LA‐ICP‐MS trace element determination

Trace element determinations by LA‐ICP‐MS were carried out in three laboratories: the State Key Laboratory of Lithospheric Evolution, IGG‐CAS, Beijing; the Geochemical Analysis Unit, CCFS/GEMOC, Macquarie University, Sydney; and the Institute of Geochemistry and Petrology, ETH Zürich. Analyses in Beijing and Zürich were done on small chips, whereas analyses in Sydney were randomly placed on the large polished slabs used for EPMA. At IGG‐CAS, a GeoLas 193 nm excimer laser was used for ablating samples, and analyses were done by means of an Agilent 7500a ICP‐MS system. The analytical details were equivalent to those described by Xie *et al*. (2008). At Macquarie University, a Photon Machines Excite 193 nm ArF excimer laser coupled to an Agilent 7700x ICP‐MS was used. The method has been described by Jackson *et al*. (2004). The ablation conditions included 50 μm beam size, 5 Hz pulse rate and 7.59 J cm^−2^ energy density. Ablation was performed in a HelEx II cell, and He was used as the carrier gas at a total flow rate of 0.825 l min^−1^. Typical measurement runs consisted of twenty analyses with four analyses of reference materials and twelve analyses of unknowns bracketed by two analyses of NIST SRM 610 (Jochum *et al*. 2011) at the beginning and end of each run. Analyses consisted of 60 s of background and 120 s of ablation. Trace element mass fractions were calculated from the raw signal data using the online software package GLITTER (Griffin *et al*. 2008). Zr was used as an internal standard to quantify trace element mass fractions, and BCR‐2 (Jochum *et al*. 2016) and NIST SRM 612 (Jochum *et al*. 2011) were used as secondary reference materials. At ETH, only GZ7 was analysed. Measurements were performed with an ASI Resolution 155 laser ablation system (193 nm ArF excimer laser) coupled to a Thermo Element XR sector‐field ICP‐MS. Data were acquired with a repetition rate of 5 Hz and an ablation spot size of 30 μm. Intensities were recorded for 70 s, which included 30 s gas blank and 40 s sample signal. The NIST SRM 612 glass was used as primary reference material, with Si used as internal standard. All data reduction and mass fraction calculations were performed using the Iolite software package (Paton *et al*. 2011).

### Titanium determination (GZ7 only)

Because of the particularly high Ti mass fraction in GZ7, this sample was subjected to precise Ti analysis by ID (isotope dilution)‐ICP‐MS at the Institute of Geochemistry and Petrology, ETH Zürich. Eleven aliquots of 1–2 mg mass were dissolved in concentrated HF in a pressure vessel (following Krogh 1973) with an addition of ^47^Ti–^49^Ti spike, repeatedly dried and redissolved in HF and HCl and subsequently evaporated to dryness. The dry residues were then dissolved in HNO_3_ with a trace of HF, and the resulting solutions were analysed using a Thermo Element XR single‐collector ICP‐MS. Details of the analyses including blank and interference corrections are described elsewhere (Szymanowski *et al*. 2018).

### Mass density determination

Mass densities were determined prior to sample preparation, by repeated weighing of the gemstones in distilled water and in air. A minute amount of detergent was added to the distilled water to decrease surface tension.

### Single‐crystal X‐ray diffraction

Unit‐cell parameters were obtained at Universität Wien by single‐crystal X‐ray diffraction analysis of small zircon chips (150–300 μm). To check for structural effects of radiation damage, chips of the natural and annealed samples were analysed. Measurements were done by means of a Huber 5042 four‐circle diffractometer, using MoKα_1,2_ radiation (λ** ~ **0.71 Å) from a conventional fine‐focus sealed tube (50 kV, 30 mA). The sample‐to‐detector distance was 420 mm. A scintillation counter with variable Soller slit was used to measure about 10–20 nonequivalent Bragg peaks for eight‐position centring according to the method of Hamilton (1974). The software SINGLE (Angel and Finger 2011) was used for diffractometer control and for calculation of lattice parameters by applying refinements with symmetry‐constraint vector least squares. The diffractometer was checked and corrected for systematic errors using the NIST SRM 1990 ruby‐sphere standard (Wong‐Ng *et al*. 2001).

### Spectroscopy

Raman and laser‐induced PL (photoluminescence) spectra were obtained at Universität Wien using a dispersive Horiba LabRAM HR Evolution system equipped with an Olympus BX41 optical microscope, a grating with 1800 grooves per mm and a Si‐based, Peltier‐cooled charge‐coupled device (CCD) detector. Point measurements were done on natural and annealed samples, to check for structural effects of radiation damage. As reference analyses, PL measurements were also done on REE^3+^‐doped ZrSiO_4_ crystals grown using a Li‐Mo flux technique (for details see Lenz *et al*. 2015, and references therein). Line scanning across the large slices was done using a software‐controlled Märzhäuser SCAN *x*–*y* stage. Raman spectra were excited using the 632.8 nm emission of a He‐Ne laser (8 mW at the sample surface). The PL spectra were excited using the 473 nm emission of a diode‐pumped solid‐state laser (5 mW at the sample surface) or a frequency‐doubled Nd:YAG laser (532 nm; 10 mW at the sample surface). The Olympus 100× objective (numerical aperture 0.9) was used. With the spectrometer system operated in full confocal mode, the lateral resolution was ~ 1 μm, and the spectral resolution was between ~ 1.5 cm^−1^ in the blue and 0.7 cm^−1^ in the NIR (near infrared) range of the electromagnetic spectrum. Wavenumber calibration was done using the Rayleigh line and Kr‐lamp emissions, resulting in a wavenumber accuracy of better than 0.5 cm^−1^. Background‐corrected spectra were fitted assuming Lorentzian–Gaussian band and line shapes. For FWHM (full width at half‐maximum) correction, the empirical formula (Váczi 2014).

(1) $$\text{FWHM}\approx {\text{FWHM}}_{\mathrm{meas}.}-{\text{IPF}}^{2}/\left(0.9\times {\text{FWHM}}_{\mathrm{meas}.}+0.1\times \text{IPF}\right)$$

has been applied, where FWHM_meas._ = measured (i.e., fitted) FWHM of the spectroscopic signal obtained, and IPF = FWHM of the instrumental profile function.

Unpolarised optical absorption spectra were obtained from the large slices of GZ7 and GZ8. Note again that slicing of the stones was done independent from the crystallographic orientation, and subjecting large fragments of the samples to the preparation of oriented slabs, for obtaining polarised spectra, would have consumed too much material. Reference measurements were done on U^4+^‐doped ZrSiO_4_ (for details see Chase and Osmer 1966) and U^5+^‐containing zircon, produced by oxidised heating of crystals from Ratanakiri, Cambodia (Zeug *et al*. 2018). Spectra were measured at room temperature in transmission geometry, by means of a Bruker IFS66v/S FTIR spectrometer equipped with a mirror‐optics IR‐scope II microscope. Circular areas of 200 μm diameter were analysed. The following combinations of light sources, beam splitters and detectors were used: Xe lamp, quartz beam splitter and GaP detector for the range 28000–19400 cm^−1^ (1024 scans; 40 cm^−1^ spectral resolution); W lamp, quartz beam splitter and Si detector for the range 19400–10000 cm^−1^ (1024 scans; 20 cm^−1^ spectral resolution); W lamp, quartz beam splitter and Ge detector for the range 10000–5250 cm^−1^ (512 scans; 10 cm^−1^ spectral resolution). Each final optical absorption spectrum hence consists of a combination of three subspectra, which were aligned to match in absorbance if necessary.

### Oxygen isotope determination

Six chips of GZ7 and seven chips of GZ8, with masses in the range 1.77–3.05 mg, were analysed for oxygen isotope ratios by laser fluorination gas source spectrometry, at the University of Wisconsin, Madison. These analyses were done in three separate sessions. All data presented in Table 3 are for chips that were analysed without any HF‐etching pretreatment (compare discussion in Valley *et al*. 2005, 2015, Nasdala *et al*. 2016). Zircon chips were heated by an infrared laser (λ = 10.6 μm) in the presence of BrF_5_. The evolved O_2_ gas was cryogenically purified, passed over hot Hg, converted to CO_2_ and analysed by means of a dual‐inlet gas source mass spectrometer that has been described elsewhere (Valley *et al*. 1994, 1995). Measured δ^18^O values were normalised to the recommended value of 5.80 VSMOW (Vienna Standard Mean Ocean Water) for the garnet reference material UWG‐2 (Valley *et al*. 1995), which was analysed six or seven times before, and two times after, analyses of GZ7 and GZ8 in each analysis session (Table 3).

### Hafnium isotope determination

Hafnium mass fractions, and ^176^Hf/^177^Hf and ^176^Lu/^177^Hf ratios, were measured by solution isotope dilution analysis of two chips each of GZ7 and GZ8 at IGG‐CAS, Beijing. After being weighed, chips were dissolved in HF‐HNO_3_ in high‐pressure bombs at 210 °C for 1 week, then dried down and dissolved again in 3 mol l^−1^ HCl. Sample solutions were then split. About 80% of each initial sample solution was used to determine the Hf isotopic composition. The remaining about 20% per solution was spiked with a mixed ^176^Lu and ^180^Hf tracer for determining the Lu and Hf mass fractions. The spike solution used was calibrated beforehand against a standard solution made from pure metals (Yang *et al*. 2010) that was tested on several calibrant materials, including BCR‐2 and W‐2 (Münker *et al*. 2001). The chemical purification procedure of Nebel‐Jacobsen *et al*. (2005) and Morel *et al*. (2008) was applied. Isotope measurements were performed on a Thermo Scientific Fisher Neptune MC‐ICP‐MS system; details of the procedure have been published elsewhere (Yang *et al*. 2010). Instrumental mass bias was corrected offline using the exponential law and assuming ^179^Hf/^177^Hf = 0.7325. Possible interferences of ^176^Yb and ^176^Lu on ^176^Hf were corrected for based on the measured ^173^Yb and ^175^Lu values, applying ^176^Lu/^175^Lu = 0.02655 and ^176^Yb/^173^Yb = 0.79631 (Vervoort *et al*. 2004). Measured ^176^Hf/^177^Hf ratios were normalised to the recommended value of 0.282160 for the Johnson Matthews Company Hf standard JMC 475 (Nowell *et al*. 1998), which was analysed in the same measurement session.

### (U‐Th)/He geochronology

(U‐Th)/He analyses were done at the University of Arizona at Tucson, to evaluate the retention of radiogenic He. Details of the experimental procedure are described elsewhere (Nasdala *et al*. 2004, Reiners 2005, Guenthner *et al*. 2016). Because the analysed aliquots were internal fragments of much larger grains, no alpha ejection correction was applied.

### U‐Pb geochronology by ID‐TIMS

The U‐Pb isotopic ratios and ages were determined by ID‐TIMS (isotope dilution–thermal ionisation mass spectrometry) in five laboratories, including NIGL (NERC Isotope Geosciences Laboratory, Keyworth, UK), University of Oslo, University of Geneva, Boise State University and Princeton University. For each of the zircon samples GZ7 and GZ8, small chips were separated from three slabs. Aliquots consisting of 5–7 fragments (with at least one fragment from each of the three slabs per sample), with total masses per aliquot in the range 2.01–2.48 mg, were given to the five ID‐TIMS laboratories for U‐Pb analysis. All laboratories were asked not to subject zircon grains to the CA (chemical abrasion) method (Mattinson 2005), in order to analyse the present U‐Pb isotope ratios and to quantify any possible postgrowth Pb loss associated with the material. Also, all laboratories were asked to report the isotopic ratios as measured, that is, without any correction for initial disequilibrium in ^230^Th/^238^U (Schärer 1984).

At each laboratory, the received fragments were broken into smaller fragments of the desired size. Zircon fragments were rinsed with some combination of distilled acetone, 6 N HCl, pure (‘Milli‐Q’) H_2_O and 3 N HNO_3_, which varied slightly depending on the laboratory. Fragments were placed in a Teflon capsule ~ 200 μl in size prior to spiking with either the EARTHTIME ET535 ^205^Pb–^233^U–^235^U tracer (Geneva, Princeton, Boise), or the ET2535 ^202^Pb–^205^Pb–^233^U–^235^U tracer (NIGL) (Condon *et al*. 2015, McLean *et al*. 2015), or a laboratory‐specific tracer (Oslo; see below). Zircon was dissolved in 29 mol l^−1^ HF + 3 mol l^−1^ HNO_3_ in pressure vessels for 60–80 h at 210–220 °C. Dissolved zircon solutions were subsequently dried down, redissolved in 6 N HCl and converted to chlorides at 185 °C overnight. U and Pb were isolated by anion exchange column chromatography AG–1 X8 resin [either Eichrom or Bio‐Rad (Krogh 1973)]. Following ion exchange chemistry, the U‐Pb aliquot was dried down with dilute (~ 0.02 mol l^−1^) H_3_PO_4_ loaded in a silica gel emitter (Gerstenberger and Haase 1997) onto an outgassed, zone‐refined Re filament for isotopic analysis. Specifics of mass spectrometry vary from laboratory to laboratory; details for each laboratory are given in online supporting information Appendix S1. Data reduction was done using a ^238^U decay constant of 1.55125 × 10^−10^ a^−1^, a ^235^U decay constant of 9.84850 × 10^−10^ a^−1^ (Jaffey *et al*. 1971) and a ^238^U/^235^U ratio of 137.82 ± 0.02 (1*s*) (Hiess *et al*. 2012).

### SIMS U‐Pb analysis

The homogeneity of the U‐Pb isotopic system of zircon samples GZ7 and GZ8, and their SIMS analysis performance (with particular attention at potential matrix effects; White and Ireland 2012), was checked by multiple analyses using the SHRIMP II (Sensitive High‐mass Resolution Ion MicroProbe) of the John de Laeter Centre for Isotopic Research, Perth. Measurements were done in two sessions, comprising 35/33/30 and 49/26/32 (GZ7/GZ8/M257) individual analyses, respectively, that were placed on a multitude of small chips embedded in an epoxy mount. Details of the instrumental conditions have been described elsewhere (Kennedy and de Laeter 1994, de Laeter and Kennedy 1998, Kennedy *et al*. 2010). The primary, mass‐filtered $O_{2}^{\text{‐}}$ beam (~ 2 nA) was focused to a ~ 15 μm elliptical spot. Data for each spot were collected through the mass range of ^196^Zr_2_O^+^, ^204^Pb^+^, background (204.1), ^206^Pb^+^, ^207^Pb^+^, ^208^Pb^+^, ^238^U^+^, ^248^ThO^+^ and ^254^UO^+^. Analyses consisted of seven cycles through these nine masses. The mass resolution, M/ΔM, was better than 5000. Results were calibrated against reference zircon M257 with an assumed ^206^Pb/^238^U age of 561.3 Ma (Nasdala *et al*. 2008).

The ^204^Pb method was used for common Pb correction (Compston *et al*. 1984, see also Ireland and Williams 2003), based on the relevant common Pb compositions from the model curve of Stacey and Kramers (1975). The high Th/U of GZ7 prevented use of the ^208^Pb common Pb correction method (Compston *et al*. 1984). The correction for instrumental Pb/U fractionation was done based on the formula of Claoué‐Long *et al*. (1995).

(2) $${}^{206}{\text{Pb}}^{+}/{}^{238}U+=a{\left({}^{238}U{}^{16}O^{+}/{}^{238}U^{+}\right)}^{b}$$

using the parameter values (*a*,* b*) of Black *et al*. (2003). Data reduction and processing were done with the Excel macro Squid 2 (Ludwig 2009). For conversion of U‐Pb isotopic ratios into ages and preparation of Wetherill Concordia diagrams (Wetherill 1956), the U decay constants of Jaffey *et al*. (1971) were used, along with the relevant common Pb compositions from the model curve of Stacey and Kramers (1975). Plotting and age calculations were done using the Excel macro Isoplot (Ludwig 2003).

## Results and discussion

### Chemical composition

**Figure 2:**
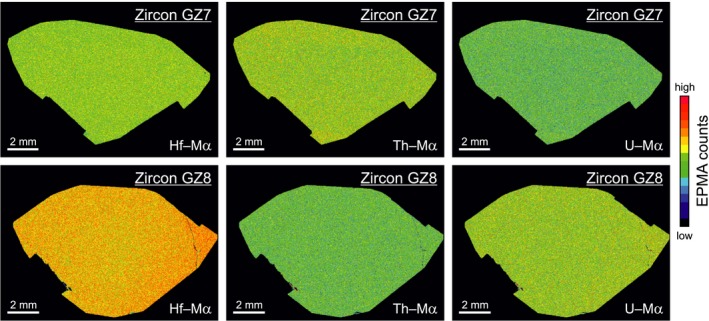
Two series of EPMA element maps of polished slabs of GZ7 and GZ8, done with uniform conditions and settings for both samples (20 kV, 3 × 10^−7^ A, dwell time 100 ms, step width 20 μm). Colour‐coded count‐rate ranges (in per s) are 260–460 for Hf, 30–102 for Th and 8–56 for U. The elongate black rectangles seen at the lower left and lower right edge (GZ7) and the lower left and upper right edge (GZ8), respectively, are terminations of Cu strips applied for improving electrical conductivity.

The element distribution within GZ7 and GZ8 appeared widely homogeneous. The BSE and CL images (not shown) and element maps (Figure 2) obtained in the EPMA did not reveal any growth zoning or other features of internal heterogeneity. Also, multiple trace element analyses in three (GZ7; *n* = 56) and two laboratories (GZ8; *n* = 28), respectively, did not show significant differences across and among the slabs and chips analysed. However, counting statistics in the EPMA element distribution maps is poor and faint differences in the trace element mass fractions are obscured by the signal noise. Quantitative EPMA line profiles across the large sample slabs (see Appendices S2 and S3) revealed slight systematic differences in U (both samples), Th (especially GZ7) and Hf (especially GZ8) mass fractions at the outer rims of the slabs. Although some systematic trend is observable, it has to be pointed out that the 2*s* errors (single point errors calculated by counting statistics) do overlap for most EPMA analysis points at each slab (see Appendix S3).

**Figure 3:**
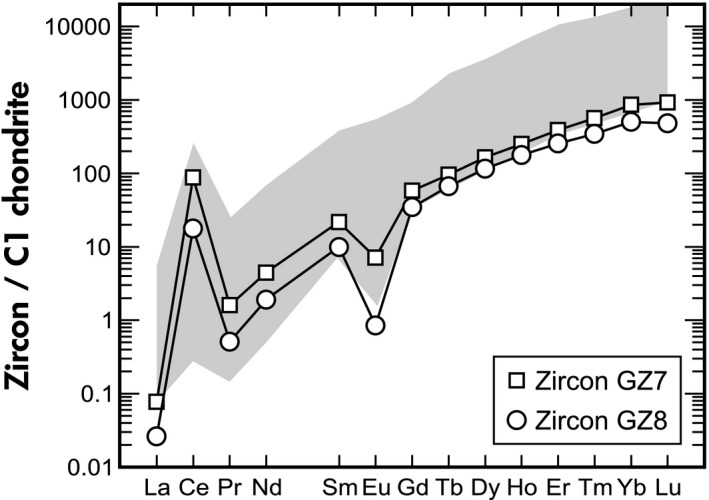
Plot of chondrite‐normalised mean REE mass fractions (LA‐ICP‐MS) measured at Chinese Academy of Sciences, Beijing, Macquarie University, Sydney, and ETH Zürich. Heights of symbols exceed 2*s* uncertainties. The grey area visualises REE mass fraction ranges of igneous zircon that were graphically extracted from figure 4 in Hoskin and Schaltegger (2003).

The EPMA and LA‐ICP‐MS Results are listed in Table 2. In general, both samples contain low levels of nonformula elements, with Hf being the only constituent with a mass fraction higher than 1%. The different Ti mass fractions (Table 2) suggest different formation temperatures for the two samples (*ca*. 830 °C for GZ7 and *ca*. 720 °C for GZ8; based on the Ti‐in‐zircon thermometer of Watson *et al*. 2006), which in turn suggests that the two samples were derived from different host rocks. The U mass fractions (GZ7, 680 μg g^−1^ ± 31 μg g^−1^; GZ8, 1305 μg g^−1^ ± 57 μg g^−1^) correspond to those of other Sri Lankan gem zircon, which typically have U mass fractions in the (0.0x–0.x)% range (Murakami *et al*. 1991, Nasdala *et al*. 2004, 2008, 2016). The U mass fraction of GZ8, however, is higher than in any other SIMS U‐Pb reference zircon. The Th/U mass fraction ratios (GZ7, 0.90; GZ8, 0.18) are significantly different, which also may indicate that the two specimens came from different source rocks. The REE (rare earth element) patterns of the two samples, in contrast, are fairly similar (Figure 3). There is a general increase in C1‐normalised mass fractions towards the heavy REE, with positive Ce anomalies and negative Eu anomalies. The slightly higher positive Ce anomaly of GZ7 (Ce/Ce* = 13.3) compared to GZ8 (Ce/Ce* = 5.86) seems to correspond well with the slightly weaker Eu anomaly of GZ7 (Eu/Eu* = 0.20) compared to GZ8 (Eu/Eu* = 0.05), both indicating somewhat more oxidising conditions in the formation of GZ7.

**Table 2:** Chemical compositions of zircon samples GZ7 and GZ8 (EPMA, Universität Göttingen; LA‐ICP‐MS, Chinese Academy of Sciences Beijing, Macquarie University Sydney and ETH Zürich)

Oxide/element	Isotope measured	Zircon GZ7	Zircon GZ8
EPMA mass fractions (%) a		(*n* = 84)	(*n* = 84)
SiO_2_	–	32.85 ± 0.08	32.53 ± 0.17
P_2_O_5_	–	0.052 ± 0.004	0.022 ± 0.003
Y_2_O_3_	–	0.078 ± 0.005	0.059 ± 0.005
ZrO_2_	–	66.40 ± 0.15	66.48 ± 0.16
Yb_2_O_3_	–	0.017 ± 0.005	0.012 ± 0.003
HfO_2_	–	1.25 ± 0.01	1.39 ± 0.02
ThO_2_	–	0.069 ± 0.005	0.027 ± 0.003
UO_2_	–	0.076 ± 0.004	0.151 ± 0.006
Total	–	100.80 ± 0.18	100.67 ± 0.29
LA‐ICP‐MS results (μg g^−1^)		(*n* = 56)	(*n* = 28)
P	31	180 ± 17	82.9 ± 12.0
Ti	49	23.8 ± 1.2	8.16 ± 1.06
Y	89	572 ± 25	436 ± 3
Nb	93	12.8 ± 0.7	8.03 ± 0.48
La	139	0.024 ± 0.006	0.008 ± 0.003
Ce	140	71.3 ± 3.9	14.3 ± 0.9
Pr	141	0.182 ± 0.032	0.057 ± 0.011
Nd	146	2.65 ± 0.37	1.14 ± 0.11
Sm	147	4.14 ± 0.40	1.90 ± 0.18
Eu	151	0.513 ± 0.067	0.061 ± 0.010
Gd	157	15.0 ± 0.9	8.96 ± 0.48
Tb	159	4.71 ± 0.19	3.27 ± 0.09
Dy	163	53.3 ± 2.3	37.5 ± 0.8
Ho	165	18.5 ± 0.7	13.1 ± 0.3
Er	166	82.6 ± 2.8	54.4 ± 1.0
Tm	169	18.0 ± 0.4	11.1 ± 0.2
Yb	173	179 ± 12	104.2 ± 4.2
Lu	175	29.8 ± 1.4	15.5 ± 0.9
Hf	178	10060 ± 290	11600 ± 240
Ta	181	3.82 ± 0.22	5.73 ± 0.38
Pb	204/206/207/208	265 ± 14	480 ± 32
Th	232	611 ± 33	240 ± 6
U	238	680 ± 31	1305 ± 57

Quoted uncertainties are 2*s*. ^a^ Al_2_O_3_, CaO and FeO were not detected or average mass fractions were below 0.005%.

**Figure 4:**
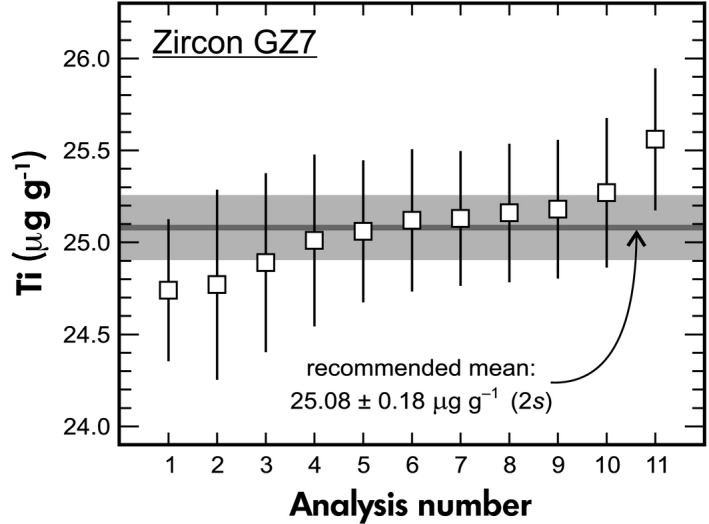
Results of eleven Ti mass fraction determinations (solution ICP‐MS) for GZ7 performed at ETH Zürich. Error bars represent 2*s* uncertainties.

The ID‐ICP‐MS analyses of GZ7 yielded uniform Ti mass fractions of 25.08 μg g^−1^ ± 0.18 μg g^−1^ (Figure 4). The Ti homogeneity in GZ7 was further supported by results of 64 LA‐ICP‐MS analyses placed at seven chips of GZ7, whose mean ^49^Ti/^29^Si ratio had a 1*s* deviation of only 1.1%. Based on these results, zircon GZ7 was proposed by Szymanowski *et al*. (2018) as reference material for analyses of Ti in zircon for the purpose of Ti‐in‐zircon geothermometry (Watson *et al*. 2006, Ferry and Watson 2007).

### Structural state

**Figure 5:**
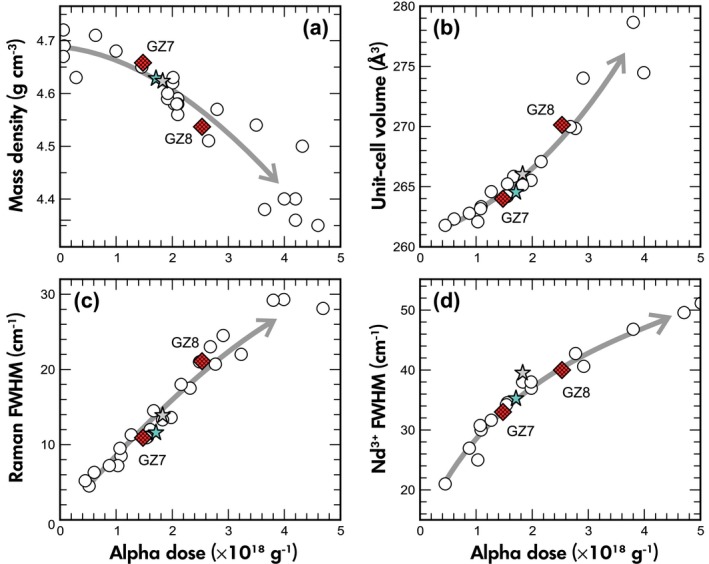
Plots of mass density (a), unit cell volume (b) and full widths at half maximum of the main Raman band at *ca*. 1000 cm^−1^ (c) and the Nd^3+^‐related emission line at *ca*. 11360 cm^−1^ (d) against the time‐integrated alpha dose, for various zircon samples from Sri Lanka. Blue star, reference zircon M257 (Nasdala *et al*. 2008). Grey star, reference zircon M127 (Nasdala *et al*. 2016). Open circles, mass densities from Vaz and Senftle (1971), Murakami *et al*. (1991), Ellsworth *et al*. (1994) and Zhang *et al*. (2000); unit‐cell volumes and Raman FWHMs from Nasdala *et al*. (2004) and unpublished data, PL FWHMs from Lenz and Nasdala (2015) and unpublished data. Grey arrows visualize the general ‘Sri Lankan’ trends of parameter changes with increasing radiation damage.

The mass densities (Table 1) were determined at 4.658 ± 0.005 g cm^−3^ (GZ7) and 4.537 ± 0.005 g cm^−3^ (GZ8), respectively. Both values coincide well with published mass densities for Sri Lankan zircon (Figure 5a), which scatter between 4.68 and 4.72 g cm^−3^ for well‐crystallised and below 4 g cm^−3^ for metamict zircon (*cf*. Holland and Gottfried 1955, Vaz and Senftle 1971, Murakami *et al*. 1991, Ellsworth *et al*. 1994, Nasdala *et al*. 2002, 2008, 2016).

Results of single‐crystal X‐ray diffraction analyses are quoted in Table 1. The unit cell of GZ7 (264.12 Å^3^ ± 0.22 Å^3^) shows moderate volume expansion and the unit cell of GZ8 (270.13 Å^3^ ± 0.20 Å^3^) shows significant volume expansion, compared to mildly radiation‐damaged zircon from Sri Lanka (~ 261 Å^3^; Holland and Gottfried 1955, Robinson *et al*. 1971, see Figure 3b). The unit‐cell expansions are consistent with the decreases in mass density. For both zircon samples, unit‐cell parameters *a*_0_ and *c*_0_ correlate with each other. This allows us to exclude any heat treatment of the gemstones, because partial annealing at comparably low temperatures would be associated with an *a*_0_–*c*_0_ mismatch (Nasdala *et al*. 2004, Chanmuang *et al*. 2017) that is explained by preferential recovery of irradiation‐induced volume swelling perpendicular to the crystallographic c axis (Weber 1990, 1993).

The FWHM of the ν_3_(SiO_4_) Raman band (internal B_1g_ mode: antisymmetric stretching of SiO_4_ tetrahedrons; Dawson *et al*. 1971) was determined at 10.9 cm^−1^ ± 0.6 cm^−1^ (GZ7) and 21.1 cm^−1^ ± 1.8 cm^−1^ (GZ8), indicating moderate and significant radiation damage, respectively (Nasdala *et al*. 1995, 2001). Multiple analyses across the large slices and of additional small chips did not yield FWHM values outside the above error ranges, indicating homogeneous structural states of both samples.

**Figure 6:**
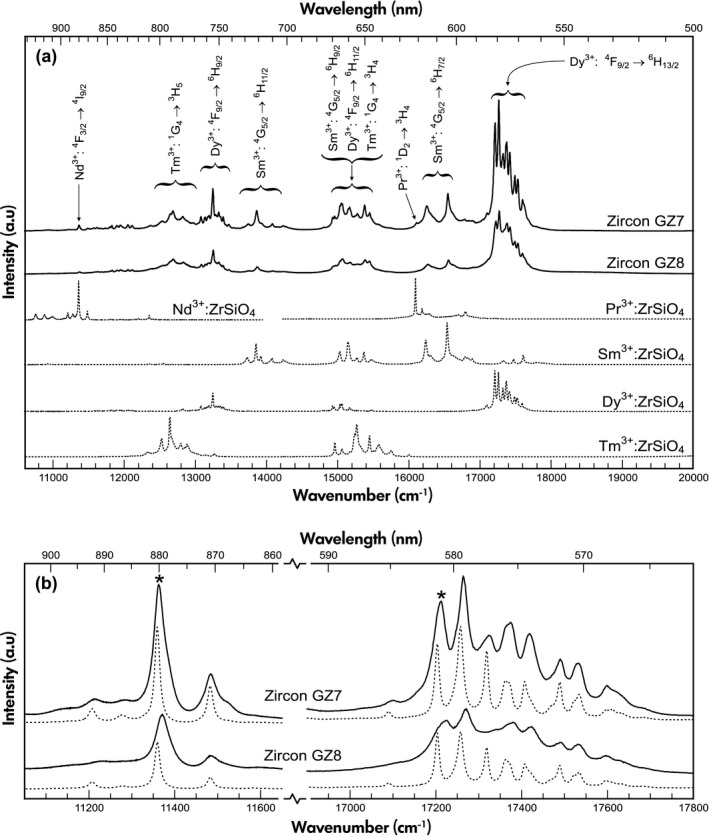
Laser‐induced PL spectroscopy. (a) Emission spectra (473 nm excitation) of GZ7 and GZ8 in comparison with reference spectra of REE‐doped ZrSiO_4_. GZ7 and GZ8 show widely similar REE‐emission patterns; the higher emission intensities of GZ7 are due to slightly higher REE mass fractions. (b) Enlargements of the ^4^F_3/2_ → ^4^I_3/2_ emission of Nd^3+^ in the near infrared range (532 nm excitation) and the ^4^F_9/2_ → ^4^H_13/2_ emission of Dy^3+^ in the green range (473 nm excitation). Spectra of untreated samples (solid) are compared with spectra obtained after structural reconstitution through annealing at 1400 °C (dotted; intensity × 0.5). Lines whose FWHMs are quoted in Table 1 are marked with asterisks.

Emission spectra (Figure 6a) do not show the yellow broadband, defect‐induced emission that typically dominates the PL of mildly radiation‐damaged zircon (Gaft *et al*. 2000, Nasdala *et al*. 2003, 2011). This indicates the presence of at least moderate defect densities, at which the yellow broadband emission is quenched already (Nasdala *et al*. 2011). The PL spectra show groups of narrow lines that are assigned to crystal‐field‐split electronic transitions of REE^3+^ (for the assignment see, e.g., Carnall *et al*. 1968, Gaft *et al*. 2000, 2015, Lenz and Nasdala 2015). The REE‐related emission intensities of GZ7 exceed in general those of GZ8 by about one‐third (Figure 6a), which corresponds to the REE mass fraction ratios of the two samples (Table 2). The fact that, in both samples, Dy^3+^ shows particularly high intensity whereas Er^3+^ and Ho^3+^ are virtually not detected, even though the mass fractions of these elements are on a similar order, is due to the different wavelength‐dependent excitation sensitivities of REE‐related emissions (Gaft *et al*. 2000, Friis *et al*. 2010, Lenz *et al*. 2015), which in turn are controlled by the particular electronic structure of each REE ion (Dieke and Crosswhite 1963, Reisfeld and Jørgensen 1977). Following Lenz and Nasdala (2015), the FWHMs of the ~ 17210 cm^−1^ sublevel of the ^4^F_9/2_ → ^4^H_13/2_ emission of Dy^3+^ and the ~ 11360 cm^−1^ sublevel of the ^4^F_3/2_ → ^4^I_9/2_ emission of Nd^3+^ (Figure 6b) were used to estimate the degree of radiation damage. These PL FWHMs are moderately (GZ7) and significantly broadened (GZ8) when compared with FWHMs of crystalline zircon (Table 1); the degrees of broadening correlate with the alpha doses (Lenz and Nasdala 2015, Figure 5d).

After reconstitution of the crystalline state through annealing at 1400 °C, the unit‐cell volumes had decreased to < 261 Å^3^ for both samples. Also, the annealed chips of GZ7 and GZ8 yielded narrow Raman bands and narrow Dy^3+^ and Nd^3+^ emission lines whose FWHMs are identical within errors to FWHMs of Raman bands and PL lines of crystalline zircon (Table 1). As references for crystalline zircon, we use the Ratanakiri, Cambodia, zircon [^206^Pb/^238^U age 0.92 ± 0.07 Ma (95% confidence uncertainty); calculated alpha dose 0.0004 × 10^18^ g^−1^; Zeug *et al*. 2018] and synthetic undoped ZrSiO_4_ (van Westrenen *et al*. 2004). Unit‐cell expansion and Raman band and PL line broadening of GZ7 and GZ8 are predominantly assigned to the accumulated radiation damage, whereas effects of minor amounts of nonformula elements on unit‐cell parameters and spectroscopic signals appear insignificant.

### Optical absorption

**Figure 7:**
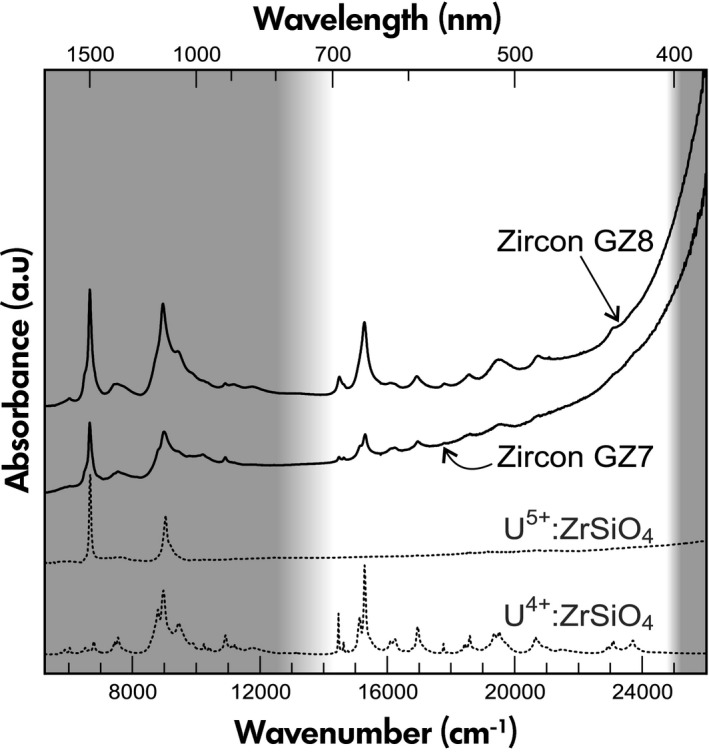
Unpolarised optical absorption spectra of GZ7 and GZ8 in comparison with reference spectra of synthetic ZrSiO_4_ doped with U^4+^ (see Chase and Osmer 1966) and U^5+^‐bearing natural zircon (Ratanakiri sample after oxidising heating; see Zeug *et al*. 2018). Spectral ranges that are invisible to the human eye are indicated by the grey background shade.

Optical absorption spectra are presented in Figure 7. In spite of their noticeably different colours, GZ7 and GZ8 yield similar principal absorption characteristics. First, an intense absorption edge that extends from the ultraviolet into the visible range and down towards the NIR region causes enhanced absorption especially of the blue fraction of the visible light. Second, there is a multitude of narrow absorption features, with the most intense at ~ 15290 cm^−1^. These are assigned to U^4+^ (Richman *et al*. 1967, Mackey *et al*. 1975) and cause absorption preferentially in the red range. The two absorption features bracket a ‘window of enhanced transmission’ in the green to yellow range that causes the yellowish green colour of GZ8. In contrast, the U^4+^ absorption of GZ7 in the red range is much less intense, which, along with a slightly different shape of the absorption edge, results in brownish colour. Both samples also are yield a fairly intense U^5+^ absorption band at ~ 6660 cm^−1^ (for assignment, see Vance and Mackey 1974), whereas the group of overlapping absorption features near ~ 8970 cm^−1^ is assigned to a combination of U^4+^ and U^5+^. The two latter, however, do not contribute to sample colouration as these absorptions are in the NIR.

The U^4+^ and U^5+^ absorption lines are significantly broadened compared to reference spectra. The effect is again assigned to radiation damage in GZ7 and GZ8. The intensity ratio of the absorption lines at ~ 15290 cm^−1^ (U^4+^) and ~ 6660 cm^−1^ (U^5+^) is notably lower for GZ7, when compared to GZ8. Although interpretations are somewhat limited, as unpolarised spectra are compared, this may indicate that GZ7 has higher U^5+^/U^4+^, which in turn seems to agree well with more oxidising conditions in the formation of GZ7 as concluded from the REE patterns.

### Further characterisation: O isotopes, Hf isotopes and (U‐Th)/He dating

Results of oxygen isotope analyses are presented in Table 3. These data yield mean δ^18^O values of 6.88‰ ± 0.05‰ VSMOW (2*s*) for GZ7 and 8.88‰ ± 0.10‰ VSMOW (2*s*) for GZ8. The significant difference of the two means strongly indicates that GZ7 and GZ8 must be derived from different host rocks. This is consistent with the different Th/U ratios and the difference in Ti mass fractions (Table 1). However, the δ^18^O values obtained for GZ7 and GZ8 do not provide independent evidence on the type of formation environment. Even though they fall well within the range of typical oxygen isotope compositions of igneous zircon (Valley *et al*. 2005), δ^18^O values of 6.88‰ and 8.88‰ VSMOW are not conclusive for igneous growth. Note that, for some homogeneous Sri Lankan reference zircon, exceptionally high δ^18^O values are reported [13.9‰ VSMOW for M257 (Nasdala *et al*. 2008); 15.4‰ VSMOW for CZ3 (Cavosie *et al*. 2011)], which rather suggest a metamorphic origin of zircon, perhaps by the metasomatic formation of skarns or similar Ca‐rich, acidic rocks from marble‐like precursors (Cavosie *et al*. 2011). This, however, does not pertain to the δ^18^O values of 6.88‰ and 8.88‰ VSMOW obtained here; there is no evidence for a skarn origin of GZ7 or GZ8, and conclusions regarding the formation environments must remain speculative. We also note that the possibility of zoning in δ^18^O or [OH] has not been determined in these crystals, either resulting from growth or alteration of more highly radiation‐damaged domains. The higher degree of radiation damage determined for GZ8 suggests that it is more likely to contain domains altered in δ^18^O. Such domains would not necessarily imply mobility of Pb or other non‐formula elements. Further information may come from *in situ* SIMS analysis of δ^18^O and [OH] by SIMS.

**Table 3:** Results of oxygen isotope analyses by laser fluorination (University of Wisconsin at Madison)

Analysis number	Sample/reference name	Material analysed	Mass (mg)	δ^18^O	
				Raw	(‰ VSMOW) a
Measurement session on 11 February 2016					
1	UWG–2	Garnet reference	4.40	5.54	
2	UWG–2	Garnet reference	2.28	5.82	
3	UWG–2	Garnet reference	2.32	5.60	
4	UWG–2	Garnet reference	1.64	5.59	
5	UWG–2	Garnet reference	1.67	5.72	
6	UWG–2	Garnet reference	1.92	5.71	
7	UWG–2	Garnet reference	2.30	5.72	
8	GZ8	Zircon	3.05	8.71	8.82
9	GZ8	Zircon	2.16	8.82	8.93
10	GZ8	Zircon	1.77	8.81	8.92
11	GZ7	Zircon	2.86	6.73	6.84
12	GZ7	Zircon	2.30	6.76	6.87
13	GZ7	Zircon	2.53	6.78	6.89
14	UWG–2	Garnet reference	2.71	5.65	
15	UWG–2	Garnet reference	3.14	5.74	
Measurement session on 20 April 2016					
1	UWG–2	Garnet reference	3.27	5.58	
2	UWG–2	Garnet reference	2.58	5.50	
3	UWG–2	Garnet reference	2.17	5.70	
4	UWG–2	Garnet reference	2.88	5.61	
5	UWG–2	Garnet reference	2.44	5.75	
6	UWG–2	Garnet reference	2.08	5.61	
7	GZ7	Zircon	2.24	6.67	6.81
8	GZ8	Zircon	2.77	8.79	8.93
9	GZ8	Zircon	2.88	8.88	9.02
10	UWG–2	Garnet reference	2.45	5.60	
11	UWG–2	Garnet reference	2.39	5.70	
Measurement session on 3 March 2017					
1	UWG–2	Garnet reference	3.19	5.45	
2	UWG–2	Garnet reference	2.86	5.47	
3	UWG–2	Garnet reference	2.30	5.42	
4	UWG–2	Garnet reference	1.72	5.39	
5	UWG–2	Garnet reference	1.50	5.45	
6	UWG–2	Garnet reference	1.74	5.44	
7	UWG–2	Garnet reference	1.54	5.46	
8	GZ7	Zircon	2.56	6.65	6.97
9	GZ7	Zircon	2.55	6.56	6.88
10	GZ8	Zircon	2.27	8.38	8.70
11	GZ8	Zircon	1.95	8.53	8.85
12	UWG–2	Garnet reference	2.19	5.60	
13	UWG–2	Garnet reference	2.29	5.49	
Summary	Zircon GZ7 (six individual analyses): Mean δ^18^O = 6.88‰ ± 0.05‰ VSMOW (2*s*)				
	Zircon GZ8 (seven individual analyses): Mean δ^18^O = 8.88‰ ± 0.10‰ VSMOW (2*s*)				

a GZ7 and GZ8 data are corrected to the respective UWG–2 reference analyses.

Results of Hf isotope analyses are listed in Table 4. Initial ^176^Hf/^177^Hf ratios and ε_Hf_(*t*) values were calculated from the measured Lu‐Hf isotopic ratios, based on a decay constant of 1.865 × 10^−11^ a^−1^ for ^176^Lu (Scherer *et al*. 2001) and the CHUR (chondritic uniform reservoir) ratios of ^176^Hf/^177^Hf of 0.282772 and ^176^Lu/^177^Hf of 0.0332 (Blichert‐Toft and Albarède 1997). Low ^176^Hf/^177^Hf ratios and hence low ε_Hf_(*t*) values of −27.7 (GZ7) and −27.4 (GZ8) indicate that both of the two zircons samples presumably have formed from reworked ancient (probably Archaean) protolith material (compare Kinny *et al*. 1991, Santosh *et al*. 2014). There is, however, no independent evidence for the formation environment. On the one hand, low εHf(*t*) values may imply metamorphic formation as reworked product of ancient crust (as discussed by Kinny *et al*. 1991). On the other hand, in rare cases igneous zircon may also yield similarly low εHf values (e.g., Yang *et al*. 2007, Wotzlaw *et al*. 2015).

**Table 4:** Results of Hf isotope determinations by ID‐ICP‐MS (Chinese Academy of Sciences Beijing)

Sample name	Lu (μg g^−1^)	Hf (μg g^−1^)	^176^Lu/^177^Hf	^176^Hf/^177^Hf	^176^Hf/^177^Hf(*t*) a	ε_Hf_(*t*) b
GZ7 #1	32.4	9323	0.00049	0.281666 ± 0.000004	0.281661	−27.7
GZ7 #2	32.8	9351	0.00050	0.281666 ± 0.000007	0.281661	−27.7
GZ8 #1	17.0	10259	0.00024	0.281662 ± 0.000005	0.281660	−27.4
GZ8 #2	17.0	10226	0.00024	0.281661 ± 0.000005	0.281659	−27.4

Quoted uncertainties of measured ^176^Hf/^177^Hf ratios are 2*s*.

a Age‐corrected (i.e., initial) ^176^Hf/^177^Hf ratios (GZ7, 530 Ma; GZ8, 544 Ma).

b ε_Hf_(*t*) = [(^176^Hf/^177^Hf(*t*)_sample_/^176^Hf/^177^Hf_CHUR_) − 1] × 104 (Faure and Mensing 2004, CHUR ^176^Hf/^177^Hf ratio from Blichert‐Toft and Albarède 1997).

Results of (U‐Th)/He analyses are summarised in Table 5. The mean He ages (2*s* uncertainties) of 438 Ma ± 3 Ma (GZ7) and 426 Ma ± 9 Ma (GZ8) fall well within the range of He ages of unheated Sri Lankan zircon (Hurley 1954, Nasdala *et al*. 2004). The He ages hence indicate that both zircon specimens have not experienced any unusual thermal history, which in turn supports that the gemstones have never been subjected to any colour enhancement through thermal treatment. The fact that He ages postdate typical U‐Pb ages of Sri Lankan gem zircon by ~ 100 Ma is explained by a prolonged cooling history of the Sri Lankan Highland Complex: After closure of the zircon U‐Pb system in the Cambrian, rocks of the Highland Complex underwent slow cooling at elevated *T* that was followed by exhumation and cooling to temperatures lower than roughly 200 °C in the Ordovician (Hölzl *et al*. 1991).

**Table 5:** (U‐Th)/He ages of GZ7 and GZ8 (University of Arizona at Tucson)

Sample name	^4^He (pmol)	U (pg)	Th (pg)	Th/U	Age (Ma)
Zircon GZ7					
16A598	1.316 ± 0.034	445 ± 6	394 ± 6	0.908	437 ± 13
16A599	0.366 ± 0.016	122 ± 2	106 ± 2	0.894	442 ± 20
16A600	1.172 ± 0.050	399 ± 6	349 ± 5	0.897	435 ± 20
16A602	0.799 ± 0.015	271 ± 4	238 ± 3	0.901	437 ± 10
Mean age of four analyses: 438 Ma ± 3 Ma (2*s*)					
Zircon GZ8					
16A603	0.563 ± 0.011	232 ± 3	42.1 ± 0.6	0.186	415 ± 10
16A604	1.670 ± 0.032	676 ± 10	119.8 ± 1.7	0.182	423 ± 10
16A605	0.758 ± 0.015	295 ± 4	52.1 ± 0.8	0.181	440 ± 11
16A606	0.655 ± 0.013	261 ± 4	47.5 ± 0.7	0.187	429 ± 10
16A607	1.235 ± 0.012	502 ± 7	88.7 ± 1.3	0.181	421 ± 7
Mean age of five analyses: 426 Ma ± 9 Ma (2*s*)					

Quoted uncertainties on individual ages are 1*s* measurement precision.

### U‐Pb geochronology results (ID‐TIMS)

**Figure 8:**
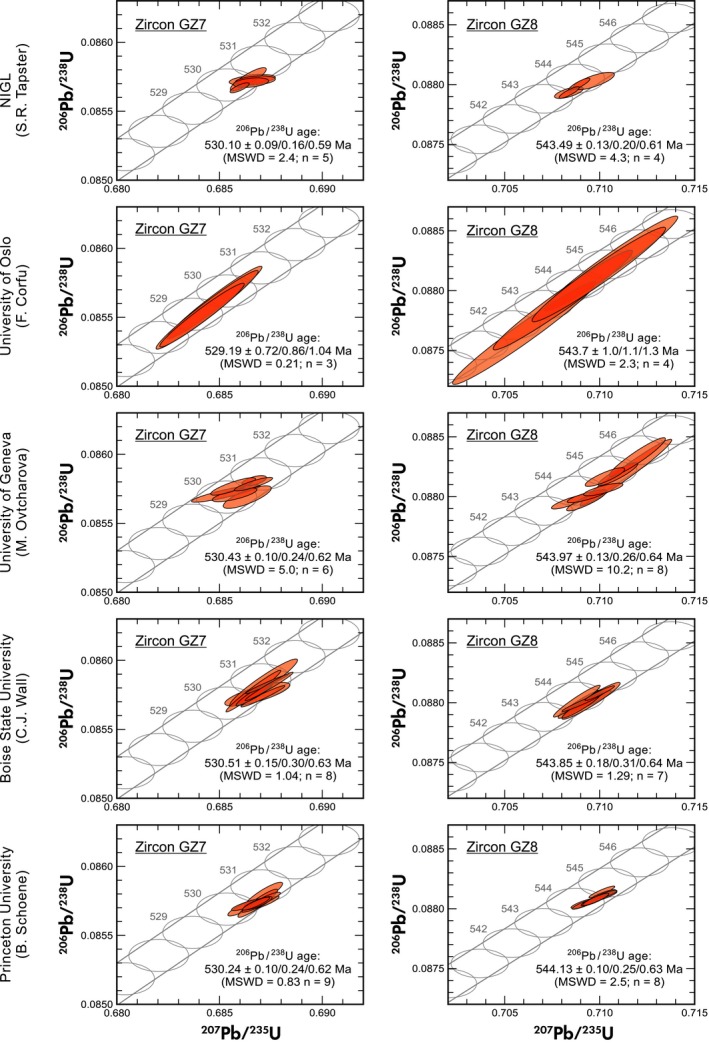
Wetherill Concordia diagrams showing results of U‐Pb isotope analyses (ID‐TIMS) performed in five laboratories. Ellipses represent 2*s*. Three uncertainties for mean ages are quoted: analytical uncertainty (2*s*) / combined analytical and tracer uncertainty / combined analytical, tracer and ^238^U decay constant uncertainty.

U‐Pb isotopic ratios and ages are listed in Tables 6 and 7. They are reported with internal errors only, including counting statistics, uncertainties in correcting for mass discrimination and the uncertainty in the common (blank) Pb composition. Wetherill Concordia plots are presented in Figure 8. Here, errors for calculated weighted mean ages quoted are of the form *x*/*y*/*z*, where *x* is solely analytical uncertainty, *y* is the combined analytical and tracer uncertainty, and *z* is the combined analytical, tracer and U decay constant uncertainty. The uncertainties in tracer calibration (0.03%; Condon *et al*. 2015, McLean *et al*. 2015) and U decay constants (0.108%; Jaffey *et al*. 1971, see also Schoene *et al*. 2006, Mattinson 2010, Boehnke and Harrison 2014) were added to the ‘internal error’ in quadrature.

**Table 6:** Results of U‐Pb determinations (ID‐TIMS) of zircon GZ7

Analysis	Compositional parameters				Isotopic ratios					Isotopic ages			
	Th/U	Pb_rad_ (pg)	Pb_com_ (pg)	Pb_rad_/Pb_com_	^206^Pb/^204^Pb	^207^Pb/^206^Pb	^207^Pb/^235^U	^206^Pb/^238^U	ρ (err. corr.)	^207^Pb/^206^Pb age (Ma)	^207^Pb/^235^U Age (Ma)	^206^Pb/^238^U Age (Ma)	Disc. (%)
(a)	(b)	(c)	(c)	(c)	(d)	(e)	(e)	(e)					(f)
NERC Isotope Geosciences Laboratory, Keyworth													
z1 (CA)	0.893	4728	1.13	4203	228816	0.05813 ± 0.00002	0.6864 ± 0.0007	0.08569 ± 0.00008	0.90	533.54 ± 1.04	530.65 ± 0.45	529.97 ± 0.48	0.67
z2 (CA)	0.909	2243	0.45	4932	267503	0.05808 ± 0.00001	0.6861 ± 0.0004	0.08571 ± 0.00004	0.82	531.74 ± 0.80	530.43 ± 0.26	530.13 ± 0.24	0.30
z3 (CA)	0.890	1773	0.54	3259	177571	0.05812 ± 0.00002	0.6856 ± 0.0007	0.08559 ± 0.00007	0.89	533.18 ± 1.09	530.14 ± 0.44	529.43 ± 0.43	0.70
z5	0.888	3262	3.14	1039	56644	0.05810 ± 0.00001	0.6860 ± 0.0004	0.08567 ± 0.00003	0.78	532.58 ± 0.77	530.38 ± 0.23	529.87 ± 0.18	0.51
z6	0.890	1503	0.50	2996	163211	0.05809 ± 0.00003	0.6864 ± 0.0007	0.08573 ± 0.00007	0.86	532.34 ± 1.19	530.64 ± 0.43	530.24 ± 0.40	0.39
z8	0.910	60.6	0.47	128	6941	0.05812 ± 0.00005	0.6865 ± 0.0007	0.08571 ± 0.00003	0.36	533.35 ± 2.11	530.71 ± 0.43	530.10 ± 0.17	0.61
z9	0.891	50.2	0.54	93	5064	0.05813 ± 0.00006	0.6867 ± 0.0008	0.08572 ± 0.00003	0.31	533.67 ± 2.42	530.84 ± 0.48	530.19 ± 0.17	0.65
z10	0.889	35.7	0.33	107	5824	0.05811 ± 0.00007	0.6866 ± 0.0009	0.08573 ± 0.00003	0.47	533.03 ± 2.64	530.75 ± 0.55	530.22 ± 0.18	0.53
University of Oslo													
464/15	0.881	40087	3.37	11890	259486	0.05804 ± 0.00004	0.6847 ± 0.0019	0.08561 ± 0.00022	0.97	530.22 ± 1.42	529.64 ± 1.14	529.50 ± 1.28	0.14
464S110 (al)	0.881	45378	3.13	14520	279916	0.05804 ± 0.00004	0.6844 ± 0.0020	0.08555 ± 0.00023	0.98	530.41 ± 1.47	529.42 ± 1.23	529.19 ± 1.39	0.24
464/16	0.882	107149	2.16	49694	179890	0.05805 ± 0.00004	0.6841 ± 0.0017	0.08552 ± 0.00019	0.97	530.62 ± 1.35	529.27 ± 1.01	528.96 ± 1.12	0.33
University of Geneva													
GZ7/z3	0.889	398	2.48	160	8733	0.05812 ± 0.00005	0.6864 ± 0.0010	0.08569 ± 0.00007	0.79	533.24 ± 2.10	530.61 ± 0.60	530.00 ± 0.40	0.61
GZ7/z2	0.905	126	0.85	149	8068	0.05805 ± 0.00006	0.6857 ± 0.0009	0.08571 ± 0.00004	0.74	530.80 ± 2.26	530.23 ± 0.55	530.10 ± 0.25	0.13
GZ7/z4	0.905	52.3	0.71	73	3976	0.05801 ± 0.00008	0.6853 ± 0.0014	0.08572 ± 0.00005	0.89	529.08 ± 3.26	529.95 ± 0.82	530.15 ± 0.30	−0.20
GZ7/z6	0.905	195	0.69	284	15387	0.05801 ± 0.00005	0.6857 ± 0.0009	0.08577 ± 0.00005	0.85	529.02 ± 1.90	530.19 ± 0.55	530.46 ± 0.31	−0.27
GZ7/z5	0.888	154	1.00	153	8332	0.05808 ± 0.00004	0.6866 ± 0.0008	0.08578 ± 0.00004	0.87	531.81 ± 1.63	530.79 ± 0.46	530.55 ± 0.24	0.24
GZ7/z7	0.904	133	0.86	153	8322	0.05807 ± 0.00003	0.6867 ± 0.0005	0.08558 ± 0.00003	0.59	531.26 ± 1.26	530.81 ± 0.29	530.70 ± 0.18	0.10
Boise State University													
z1	0.895	753	14.3	53	2866	0.05811 ± 0.00008	0.6872 ± 0.0013	0.08576 ± 0.00007	0.78	534.08 ± 2.97	531.09 ± 0.78	530.39 ± 0.42	0.69
z2	0.898	2281	1.41	1617	87638	0.05815 ± 0.00003	0.6874 ± 0.0009	0.08574 ± 0.00006	0.97	535.33 ± 1.29	531.26 ± 0.53	530.32 ± 0.38	0.94
z3	0.891	976	2.05	477	25928	0.05808 ± 0.00004	0.6875 ± 0.0013	0.08584 ± 0.00014	0.95	532.91 ± 1.46	531.28 ± 0.81	530.90 ± 0.83	0.38
z4	0.898	2776	0.38	7316	396417	0.05808 ± 0.00002	0.6870 ± 0.0011	0.08578 ± 0.00012	0.98	532.88 ± 0.79	530.99 ± 0.66	530.55 ± 0.73	0.44
z5	0.915	813	0.75	1085	58582	0.05811 ± 0.00003	0.6870 ± 0.0007	0.08576 ± 0.00006	0.93	533.73 ± 0.96	531.02 ± 0.41	530.39 ± 0.35	0.63
z6	0.915	1128	0.30	3796	204903	0.05810 ± 0.00002	0.6870 ± 0.0007	0.08576 ± 0.00007	0.95	533.60 ± 0.88	531.02 ± 0.45	530.42 ± 0.43	0.60
z7	0.892	437	0.25	1777	96454	0.05810 ± 0.00003	0.6876 ± 0.0008	0.08583 ± 0.00006	0.98	533.68 ± 1.31	531.36 ± 0.51	530.82 ± 0.34	0.54
z8	0.914	407	0.33	1241	67017	0.05812 ± 0.00003	0.6877 ± 0.0010	0.08581 ± 0.00008	0.98	534.47 ± 1.04	531.43 ± 0.58	530.72 ± 0.50	0.70
Princeton University													
z1	0.897	842	0.50	1680	90671	0.05816 ± 0.00002	0.6873 ± 0.0006	0.08574 ± 0.00005	0.88	534.86 ± 1.17	531.15 ± 0.39	530.29 ± 0.27	0.85
z2	0.898	596	0.38	1566	84504	0.05815 ± 0.00002	0.6873 ± 0.0007	0.08577 ± 0.00005	0.88	534.35 ± 1.17	531.18 ± 0.39	530.44 ± 0.28	0.73
z3	0.897	546	0.36	1522	82125	0.05813 ± 0.00003	0.6868 ± 0.0007	0.08573 ± 0.00005	0.88	533.57 ± 1.19	530.86 ± 0.40	530.23 ± 0.27	0.63
z4	0.897	1052	0.34	3130	168926	0.05814 ± 0.00002	0.6867 ± 0.0006	0.08571 ± 0.00004	0.90	533.93 ± 1.14	530.83 ± 0.38	530.11 ± 0.25	0.72
z5	0.897	220	0.56	390	21070	0.05810 ± 0.00004	0.6864 ± 0.0008	0.08572 ± 0.00004	0.86	532.40 ± 1.63	530.61 ± 0.46	530.19 ± 0.25	0.42
z6	0.897	419	0.47	885	47797	0.05809 ± 0.00003	0.6861 ± 0.0007	0.08570 ± 0.00006	0.87	532.31 ± 1.29	530.48 ± 0.44	530.05 ± 0.35	0.42
z7	0.896	302	0.43	694	37506	0.05813 ± 0.00003	0.6869 ± 0.0007	0.08573 ± 0.00005	0.83	533.76 ± 1.41	530.91 ± 0.42	530.25 ± 0.29	0.66
z9	0.897	623	1.06	590	31860	0.05811 ± 0.00003	0.6872 ± 0.0009	0.08580 ± 0.00009	0.89	533.08 ± 1.37	531.09 ± 0.55	530.62 ± 0.53	0.46
z10	0.897	1105	0.22	4966	267986	0.05813 ± 0.00002	0.6869 ± 0.0010	0.08573 ± 0.00010	0.93	533.75 ± 1.17	530.91 ± 0.58	530.25 ± 0.60	0.65
Summary: Means of 31 individual analyses						^206^Pb/^238^U = 0.085735 ± 0.000009 (2*s*); ^206^Pb/^238^U age = 530.26 Ma ± 0.05 Ma (95% confidence; MSWD 3.1)							

Quoted uncertainties are 2*s*. a, Three analyses marked ‘(CA)’ were done after chemical abrasion following Mattinson (2005); these results were disregarded in the calculation of mean isotopic ratios and ages. The analysis marked ‘(al)’ was done on an aliquot of 464/15. b, Model Th/U ratios were calculated from the radiogenic ^208^Pb and the ^230^Th‐corrected ^206^Pb/^238^U age. c, Pb_rad_ = total mass of radiogenic Pb; Pb_com_ = total mass of common Pb. d, Measured ^206^Pb/^204^Pb ratio corrected for fractionation and spike contribution only. e, Measured ratios corrected for fractionation, tracer and blank. f, Discordance = 100 − [100 × (^206^Pb/^238^U date)/(^207^Pb/^206^Pb date)].

**Table 7:** Results of U‐Pb determinations (ID‐TIMS) of zircon GZ8

Analysis	Compositional parameters				Isotopic ratios					Isotopic ages			
	Th/U	Pb_rad_ (pg)	Pb_com_ (pg)	Pb_rad_/Pb_com_	^206^Pb/^204^Pb	^207^Pb/^206^Pb	^207^Pb/^235^U	^206^Pb/^238^U	ρ (err. corr.)	^207^Pb/^206^Pb age (Ma)	^207^Pb/^235^U Age (Ma)	^206^Pb/^238^U Age (Ma)	Disc. (%)
(a)	(b)	(c)	(c)	(c)	(d)	(e)	(e)	(e)					(f)
NERC Isotope Geosciences Laboratory, Keyworth													
z1 (CA)	0.189	4029	1.00	4040	263427	0.05846 ± 0.00001	0.7088 ± 0.0006	0.08798 ± 0.00006	0.92	545.96 ± 0.83	544.04 ± 0.38	543.58 ± 0.37	0.44
z2 (CA)	0.189	6237	0.53	11774	767738	0.05844 ± 0.00001	0.7086 ± 0.0006	0.08798 ± 0.00006	0.92	545.29 ± 0.73	543.89 ± 0.35	543.56 ± 0.37	0.32
z3 (CA)	0.188	2985	1.59	1878	122508	0.05846 ± 0.00002	0.7088 ± 0.0005	0.08798 ± 0.00005	0.87	546.18 ± 0.86	544.06 ± 0.32	543.55 ± 0.30	0.48
z5	0.189	4369	0.75	5859	382041	0.05845 ± 0.00001	0.7089 ± 0.0005	0.08800 ± 0.00004	0.87	545.71 ± 0.72	544.10 ± 0.28	543.71 ± 0.25	0.37
z6	0.185	3417	2.10	1624	106018	0.05845 ± 0.00001	0.7084 ± 0.0004	0.08793 ± 0.00004	0.85	545.80 ± 0.71	543.79 ± 0.26	543.31 ± 0.24	0.46
z8	0.188	149	0.30	495	32317	0.05845 ± 0.00003	0.7084 ± 0.0006	0.08793 ± 0.00004	0.68	545.79 ± 1.44	543.78 ± 0.37	543.30 ± 0.24	0.46
z10	0.189	212	0.95	224	14640	0.05850 ± 0.00005	0.7097 ± 0.0009	0.08803 ± 0.00006	0.73	547.42 ± 1.90	544.56 ± 0.52	543.88 ± 0.35	0.65
University of Oslo													
464/13	0.180	59546	7.33	8121	383539	0.05845 ± 0.00006	0.7099 ± 0.0029	0.08814 ± 0.00032	0.97	545.61 ± 3.16	544.72 ± 1.71	544.50 ± 1.91	0.21
464S104 (al)	0.180	59315	7.39	8025	380177	0.05844 ± 0.00006	0.7106 ± 0.0029	0.08823 ± 0.00032	0.97	545.33 ± 3.15	545.11 ± 1.71	545.11 ± 1.91	0.05
464/14	0.182	54721	8.26	6622	326006	0.05845 ± 0.00006	0.7060 ± 0.0031	0.08765 ± 0.00035	0.97	545.63 ± 3.17	542.37 ± 1.84	541.60 ± 2.09	0.77
464/S107 (al)	0.180	57792	7.05	8196	381192	0.05843 ± 0.00006	0.7080 ± 0.0030	0.08792 ± 0.00034	0.97	545.08 ± 3.15	543.59 ± 1.78	543.23 ± 2.01	0.35
University of Geneva													
GZ8/z6	0.184	141	0.79	179	11658	0.05844 ± 0.00004	0.7084 ± 0.0008	0.08796 ± 0.00005	0.85	545.37 ± 1.56	543.81 ± 0.47	543.44 ± 0.29	0.35
GZ8/z2	0.185	2680	1.88	1426	92860	0.05851 ± 0.00003	0.7093 ± 0.0009	0.08796 ± 0.00007	0.90	547.95 ± 1.28	544.34 ± 0.49	543.48 ± 0.40	0.82
GZ8/z7	0.184	230	0.86	268	17456	0.05848 ± 0.00004	0.7093 ± 0.0009	0.08800 ± 0.00004	0.85	546.99 ± 1.78	544.35 ± 0.51	543.72 ± 0.26	0.60
GZ8/z8	0.189	65.3	0.81	81	5255	0.05852 ± 0.00005	0.7102 ± 0.0009	0.08806 ± 0.00004	0.80	548.29 ± 1.95	544.87 ± 0.52	544.05 ± 0.25	0.77
GZ8/z5	0.189	312	0.85	368	23970	0.05846 ± 0.00004	0.7103 ± 0.0009	0.08816 ± 0.00006	0.86	545.97 ± 1.49	544.92 ± 0.50	544.67 ± 0.38	0.24
GZ8/z3	0.189	2330	0.86	2719	176799	0.05850 ± 0.00003	0.7110 ± 0.0009	0.08819 ± 0.00008	0.90	547.44 ± 1.27	545.32 ± 0.52	544.81 ± 0.46	0.48
GZ8/z4	0.189	3330	0.88	3785	246043	0.05852 ± 0.00003	0.7114 ± 0.0020	0.08820 ± 0.00023	0.98	548.46 ± 1.34	545.60 ± 1.18	544.90 ± 1.38	0.66
GZ8/z1	0.189	2580	0.92	2802	182215	0.05848 ± 0.00003	0.7120 ± 0.0012	0.08833 ± 0.00013	0.94	546.99 ± 1.28	545.92 ± 0.73	545.67 ± 0.78	0.24
Boise State University													
z1	0.184	1570	0.54	2918	187944	0.05846 ± 0.00003	0.7093 ± 0.0009	0.08799 ± 0.00010	0.95	547.23 ± 0.98	544.34 ± 0.55	543.65 ± 0.57	0.66
z2	0.184	696	0.32	2155	140317	0.05849 ± 0.00002	0.7104 ± 0.0006	0.08808 ± 0.00005	0.95	548.20 ± 0.86	544.96 ± 0.38	544.18 ± 0.30	0.73
z3	0.188	1169	0.24	4831	314142	0.05849 ± 0.00004	0.7098 ± 0.0010	0.08802 ± 0.00007	0.93	548.10 ± 1.46	544.62 ± 0.57	543.79 ± 0.42	0.79
z4	0.188	1882	0.49	3872	251773	0.05850 ± 0.00004	0.7099 ± 0.0013	0.08802 ± 0.00013	0.95	548.38 ± 1.32	544.70 ± 0.75	543.83 ± 0.75	0.83
z5	0.184	753	0.48	4703	306182	0.05847 ± 0.00003	0.7095 ± 0.0009	0.08801 ± 0.00009	0.97	547.32 ± 1.27	544.44 ± 0.53	543.75 ± 0.37	0.65
z6	0.188	1866	0.23	8085	525776	0.05844 ± 0.00002	0.7092 ± 0.0010	0.08802 ± 0.00010	0.96	546.11 ± 0.91	544.26 ± 0.60	543.82 ± 0.65	0.42
z7	0.184	2077	0.27	7835	509976	0.05848 ± 0.00002	0.7094 ± 0.0008	0.08798 ± 0.00008	0.95	547.86 ± 0.90	544.40 ± 0.49	543.58 ± 0.49	0.78
Princeton University													
z1	0.188	574	0.43	1321	85485	0.05849 ± 0.00003	0.7102 ± 0.0007	0.08810 ± 0.00004	0.88	547.21 ± 1.21	544.86 ± 0.39	544.30 ± 0.25	0.53
z2	0.188	352	0.39	913	59074	0.05850 ± 0.00003	0.7103 ± 0.0007	0.08810 ± 0.00004	0.90	547.55 ± 1.19	544.90 ± 0.39	544.27 ± 0.24	0.60
z3	0.189	252	0.49	518	33525	0.05846 ± 0.00003	0.7092 ± 0.0007	0.08803 ± 0.00004	0.86	545.91 ± 1.33	544.26 ± 0.41	543.87 ± 0.25	0.37
z4	0.188	456	0.52	870	56301	0.05846 ± 0.00003	0.7092 ± 0.0007	0.08802 ± 0.00005	0.89	546.16 ± 1.21	544.29 ± 0.41	543.84 ± 0.27	0.42
z5	0.188	1134	0.45	2543	164470	0.05848 ± 0.00002	0.7098 ± 0.0007	0.08806 ± 0.00005	0.89	547.00 ± 1.14	544.63 ± 0.41	544.06 ± 0.31	0.54
z6	0.188	706	0.36	1982	128214	0.05847 ± 0.00002	0.7101 ± 0.0007	0.08812 ± 0.00005	0.92	546.42 ± 1.08	544.81 ± 0.40	544.43 ± 0.29	0.36
z7	0.189	488	0.53	916	59261	0.05850 ± 0.00003	0.7103 ± 0.0007	0.08809 ± 0.00004	0.88	547.74 ± 1.21	544.90 ± 0.39	544.22 ± 0.25	0.64
z8	0.188	709	0.61	1165	75402	0.05848 ± 0.00003	0.7097 ± 0.0007	0.08806 ± 0.00005	0.88	546.87 ± 1.19	544.57 ± 0.41	544.03 ± 0.29	0.52
Summary: Means of 31 individual analyses						^206^Pb/^238^U = 0.088037 ± 0.000010 (2*s*); ^206^Pb/^238^U age = 543.92 Ma ± 0.06 Ma (95% confidence; MSWD 6.0)							

Quoted uncertainties are 2*s*. a, Three analyses marked ‘(CA)’ were done after chemical abrasion following Mattinson (2005); these results were disregarded in the calculation of mean isotopic ratios and ages. The two analyses marked ‘(al)’ were done on aliquots of 464/13 and 464/14, respectively. b, Model Th/U ratios were calculated from the radiogenic ^208^Pb and the ^230^Th‐corrected ^206^Pb/^238^U age. c, Pb_rad_ = total mass of radiogenic Pb; Pb_com_ = total mass of common Pb. d, Measured ^206^Pb/^204^Pb ratio corrected for fractionation and spike contribution only. e, Measured ratios corrected for fractionation, tracer and blank. f, Discordance = 100 − [100 × (^206^Pb/^238^U date)/(^207^Pb/^206^Pb date)].

A total of thirty‐one ID‐TIMS analyses without prior CA treatment were done for each of the two zircon samples GZ7 and GZ8. The recommended mean ^206^Pb/^238^U values are 0.085735 ± 0.000009 (2*s*) for GZ7 and 0.088037 ± 0.000010 (2*s*) for GZ8. The weighted mean ^206^Pb/^238^U ages (uncertainties quoted at the 95% confidence level) are 530.26 Ma ± 0.05 Ma (MSWD 3.1) for GZ7 and 543.92 Ma ± 0.06 Ma (MSWD 6.0) for GZ8. Both of these ages are concordant within the uncertainties of decay constants. It should be noted that at NIGL, three additional ID‐TIMS analyses of each zircon sample were done that were preceded by CA treatment according to Mattinson (2005). The results are included in Tables 6 and 7; however, they were disregarded in the calculation of mean isotopic ratios and ages. Systematic deviations of the results (isotopic ratios and degrees of U‐Pb discordance) from those of analyses without CA were not observed.

The ~ 14 Ma difference between the two U‐Pb dates is not unusual for gem zircon from the Sri Lankan Highland Complex. Published ages scatter in the approximate range 575–520 Ma (Pidgeon *et al*. 1994, Claoué‐Long *et al*. 1995, Kennedy 2000, Stern 2001, Nasdala *et al*. 2004, 2008, 2016). However, the age difference supports again that GZ7 and GZ8 were derived from different source rocks.

### SIMS U‐Pb analysis

**Figure 9:**
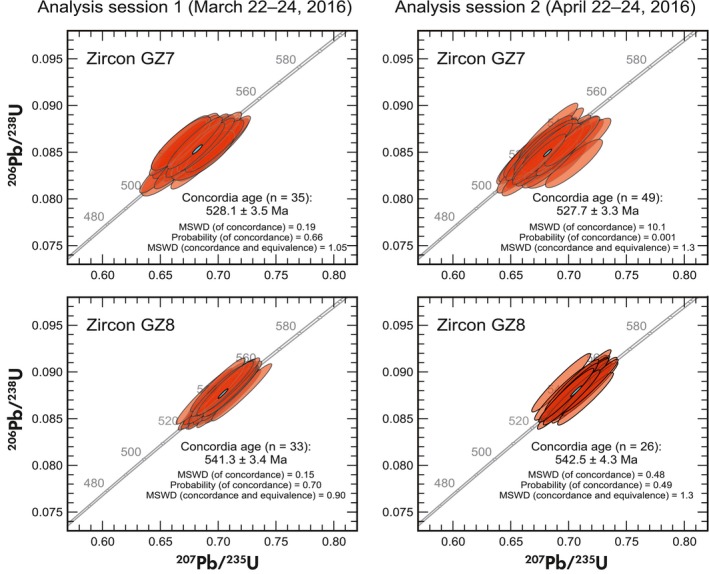
Wetherill Concordia diagrams showing results of U‐Pb analyses (SIMS) performed at Curtin University, Perth. Error ellipses represent 1*s* uncertainties. Results were calibrated versus M257 with an assumed ^206^Pb/^238^U age of 561.3 Ma (Nasdala *et al*. 2008). Concordia ages are quoted at the 95% confidence level and include uncertainties of decay constants.

Results of SIMS analyses are presented in Figure 9 and given in Appendix S4 (which contains data, additional Concordia diagrams and plots of Th/U for GZ7 and GZ8). Zircon GZ8 exhibits somewhat variable Th/U, with a single chip having Th/U = 0.1872 ± 0.0004 (*n* = 13), which is significantly higher than all other chips, which have Th/U = 0.1816 ± 0.0002 (*n* = 46). There is no correlation between Th/U and the U‐Pb isotopic ratios obtained. Based on the EPMA line scans, we may speculate that this chip originated from a rim area of the initial stone (see Appendix S3).

The calculated mean Concordia ages (Ludwig 1998) given in Figure 9 coincide within errors with the ID‐TIMS results, even though they seem to be slightly (*ca*. 2 Ma) younger. It may be speculated that the apparently systematic bias is caused by the particular data reduction calibration parameters. For instance, applying ^208^Pb correction to the same SIMS data for zircon GZ8 results in a mean Concordia age of 546.4 Ma ± 1.3 Ma (1*s*), which is 2.5 Ma older than the ID‐TIMS result. However, the apparent age differences are below the reproducibility of SIMS results (typically ~ 1%). It nevertheless seems worthwhile that more SIMS laboratories check whether there is a systematic bias between SIMS and ID‐TIMS results, prior to using GZ7 and GZ8 as reference materials.

Two important observations can be made from the SIMS results. First, both zircon samples did not reveal any detectable heterogeneity of the U‐Pb isotopic ratios within and between the sessions. Second, even though zircon GZ8 is significantly more radiation‐damaged than M257 and any other SIMS reference, there were no noticeable matrix effects under the $O_{2}^{\text{‐}}$ beam. Too high levels of radiation damage can effectuate systematically enhanced emission of Pb^+^ relative to U and U oxide species, which would result in reversely discordant U‐Pb isotopic ratios (White and Ireland 2012). This has not been observed, suggesting that the sputter behaviour under the $O_{2}^{\text{‐}}$ beam of both GZ7 and GZ8 does not cause systematically different ion yields to that of unknowns, which in turn is most promising in terms of the performance as reference materials.

## Concluding remarks

Zircon samples GZ7 and GZ8 constitute suitable reference materials for the U‐Pb analysis of unknown zircon samples by means of SIMS. Both reference materials are isotopically homogeneous and have a concordant U‐Pb system, low levels of non‐radiogenic Pb and comparably high U and Pb mass fraction. The latter are expected to result in high count rates and good Poisson statistics during analysis. Both reference materials did not show noticeable matrix effects (that is, preferred sputtering of Pb isotopes resulting in reverse discordance) under the $O_{2}^{\text{‐}}$ beam. Features pointing to a postgrowth chemical alteration history have not been found, and our measurement results allow us to exclude any unusual thermal history. This also applies to the common practice of Sri Lankan gem miners and dealers to enhance colour and clarity of zircon specimens by heating them in an open fire, which can be excluded in the case of GZ7 and GZ8.

More than 3500 mg are still available for each of the samples GZ7 and GZ8. They will be distributed and made available for SIMS U‐Pb analysis. A major fraction of the material will be used and distributed for SIMS analytical work in other laboratories, by the Beijing SHRIMP Centre, Institute of Geology, Chinese Academy of Geological Sciences (contact: liudunyi@bjshrimp.cn). However, it needs to be emphasised that samples will *not* be provided for LA‐ICP‐MS U‐Pb geochronology. This explicit decision is made to reduce the consumption of the two reference materials to a minimum. We wish to ensure that the materials will be available for SIMS work for a long period.

## Supporting information

This material is available as part of the online article from: http://onlinelibrary.wiley.com/doi/10.1111/ggr.12239/abstract (This link will take you to the article abstract).

Appendix S1. Details for ID‐TIMS analytical procedures in the laboratories.

Appendix S2. Measurement results from EPMA (*n* = 84 for each of the two zircon samples) at Universität Göttingen, Germany.

Appendix S3. Documentation of locations of EPMA (Universität Göttingen, Germany) linescans, and plots and histograms of mass fractions of HfO_2_, ThO_2_ and UO_2_.

Appendix S4. Measurement results from SHRIMP analyses (Curtin University, Perth, Australia) including additional Concordia plots and Th/U histograms.
