# Supplementary material for: GZ7 and GZ8 – Two Zircon Reference Materials for SIMS U‐Pb Geochronology
Source: Geostand Geoanal Res. 2018 Oct 8;42(4):431–57. doi: 10.1111/ggr.12239 (PMC6334521; doi:10.1111/ggr.12239)
Supplement: Supplementary file 1 — Appendix S1. Details for ID‐TIMS analytical procedures in the laboratories. [file GGR-42-431-s001.pdf]

Supplement 1.
Details for analytical procedures in the five ID-TIMS laboratories

ID–TIMS analyses at NIGL

Isotope ratio measurements were made using a Thermo Scientific Triton thermal ionization mass-spectrometer. Lead was either measured in peak hopping (dynamic) mode on a single MasCom secondary electron multiplier (SEM) for lower beam intensities, or more typically, by a dynamic combined Faraday-SEM peak hopping mode whereby mass 205 was cycled with masses 201, 203 and 204 in the axial SEM, and measured simultaneously with either mass 202 and 205, or masses 205–208 in Faraday cups with 10^{11} Ω resistors. Linearity and dead time corrections on the SEM were monitored using repeated analyses of the NBS 982, NBS 981 (Catanzaro *et al.* 1968) and CRM U500 (Condon *et al.* 2010) standards. Uranium was run as an oxide (UO_2) and measured in static mode on Faraday detectors equipped with 10^{12} Ω resistors. Raw U and Pb data were filtered using the Tripoli software program (Bowring *et al.* 2011). The Pb and U mass fractionation was calculated in real-time based on the isotopic composition of the double spiked ET2535 (v. 3.0) tracer. Uranium oxide measurements were corrected for isobaric interferences using an $^{18}O/^{16}O$ value of 0.00205. The Pb measurements were corrected for isobaric interferences of $BaPO_3$ and Tl using the monitoring masses of 201 and 203. Data reduction, date calculation/presentation and uncertainty propagation were done in ET_Redux (McLean *et al.* 2011). Uranium blanks were assumed to be 0.1 pg ± 0.01 pg (1*s*). Common Pb was attributed entirely to laboratory blank using a calculated Pb isotopic composition for the laboratory blank of $^{206}Pb/^{204}Pb$ = 18.10 ± 0.05; $^{207}Pb/^{204}Pb$ = 15.55 ± 0.02 and $^{208}Pb/^{204}Pb$ = 37.82 ± 0.16 (1*s* uncertainties). Note that at NIGL, several additional analyses were subjected to chemical abrasion (CA-ID-TIMS) for comparison, and that procedure used an abridged approach following Mattinson (2005).

ID–TIMS analyses at University of Oslo

Zircon fragments were first cleaned with HNO_3, H_2O and acetone, and then weighed on a microbalance. Fragments were transferred to Krogh-type Teflon bombs and dissolved in HF at 195 °C after adding a $^{202}Pb–^{205}Pb–^{235}U$ spike. Analysis of the Pb and U isotope compositions was done by means of a Finnigan MAT262 mass spectrometer. Further analytical details are documented elsewhere (Corfu 2004). All isotope ratios and ages were corrected for fractionation, spike, blank ($^{206}Pb/^{204}Pb$ = 18.3; $^{207}Pb/^{204}Pb$ = 15.555) and initial common Pb (based on Stacey and Kramers 1975). Errors were calculated by quadratic propagation of the main sources of uncertainty using an in-house program. The U–Pb ratio of the spike used is adapted to $^{206}Pb/^{238}U$ = 0.015660 for the EARTHTIME ET100 solution, as obtained with the EARTHTIME ET2535 spike (Condon *et al.* 2015, McLean *et al.* 2015) at the NERC Isotope Geosciences Laboratory.

ID–TIMS analyses at University of Geneva

Isotopic analyses were performed on a Thermo Scientific Triton mass spectrometer equipped with a MasCom discrete dynode electron multiplier. The linearity of the multiplier was calibrated using CRM U500 (Condon *et al.* 2010), NBS 982 and NBS 983 (Catanzaro *et al.* 1968) solutions. The deadtime for the SEM was determined to be constant at 22.5 ns for up to a count rate of 1.3×10^6 s^{-1} and at a Faraday/SEM yield between 93 % and 94 %. During the time of the measurements isobaric interferences from $BaPO_2^+$ or Tl^+ were monitored by measuring masses 201 and 203 in spiked, and masses 202 and 205 in unspiked samples. Since no statistically significant signal was observed on the controlled masses, no correction was applied. Lead-isotope fractionation was corrected based on average Pb fractionation factors determined by EARTHTIME ^{202}Pb–^{205}Pb–^{233}U–^{235}U tracer and measurements of NBS 981 (Catanzaro *et al.* 1968) standard (0.13 % amu^{-1} ± 0.02 % amu^{-1}). The U mass fractionation for the same analyses was calculated using the ^{233}U/^{235}U ratio of the double spike solution (0.99506 ± 0.01 %, 1*s*). The average U fractionation factor was 0.08 % amu^{-1} ± 0.02 % amu^{-1} (1*s*). Both Pb and U were loaded with 1 µl of silica gel–phosphoric acid mixture (modified after Gerstenberger and Haase, 1997) on outgassed single Re filaments. Lead-isotope compositions were measured on the electron multiplier, while U (as UO_2) isotopic measurements were made in static Faraday mode (using amplifiers equipped with 10^{12} Ω resistors). Isobaric interference of $^{233}U^{18}O^{16}O$ on $^{235}U^{16}O^{16}O$ was corrected using an $^{18}O/^{16}O$ ratio of 0.00205.

All common Pb in the zircon analyses was attributed to the procedural blank with the following Pb isotopic composition: ^{206}Pb/^{204}Pb = 18.18 ± 0.12; ^{207}Pb/^{204}Pb = 15.50 ± 0.05; ^{208}Pb/^{204}Pb = 37.58 ± 0.16 (1*s*). Uranium blanks were <0.1 pg and did not influence the degree of discordance at the age range of the studied samples, therefore a value of 0.0005 pg (±50 %) was used in all data reduction. The initial statistics, data reduction and age calculation were done using the TRIPOLI and Redux software (Bowring *et al.* 2011). The accuracy of the measured data was assessed by repeated analysis of the EARTHTIME ET100 synthetic solution (Condon *et al.* 2008) yielding an internal reproducibility in ^{206}Pb/^{238}U dates of better than 0.05 %. The ET100 synthetic solution measured with EARTHTIME ^{202}Pb–^{205}Pb–^{235}U–^{238}U tracer yielded a mean ^{206}Pb/^{238}U ratio of 100.249 Ma ± 0.014 Ma (MSWD = 1.6; n = 10).

ID–TIMS analyses at Boise State University

Isotopic determinations were performed using an IsotopX PhoeniX-62 TIMS. A correction for mass-dependent Pb fractionation was applied based on repeated measurements of NBS 982 (Catanzaro *et al.* 1968) Pb [on both the Daly ion counter [0.16 ± 0.03 %) amu^{-1}; 1*s*] and the Faraday cups [0.10 × (1 ± 0.02 %) amu^{-1}; 1*s*]. Uranium was run as an oxide (UO_2) and measured in static mode on Faraday detectors equipped with 10^{12} Ω resistors. The U mass fractionation for the same analyses was calculated using the ^{233}U/^{235}U ratio of the double spike solution (0.99506 ± 0.01 %, 1*s*). Raw U and Pb data were filtered using the Tripoli software program (Bowring *et al.* 2011) and the U-Pb dates and

uncertainties for each analysis were calculated using the algorithms of Schmitz and Schoene (2007). Uranium oxide measurements were corrected for isobaric interferences using an $^{18}O/^{16}O$ value of 0.00206. More analytical details are reported elsewhere (Davydov *et al.* 2010, Schmitz and Davydov 2012).

Uncertainties are based upon non-systematic analytical errors, including counting statistics, instrumental fractionation, tracer subtraction, and blank subtraction. All non-radiogenic Pb was attributed to laboratory blank with a mean isotopic composition determined by total procedural blank measurements. These error estimates should be considered when comparing the $^{206}Pb/^{238}U$ dates with those from other laboratories that used tracer solutions calibrated against the EARTHTIME gravimetric standards.

ID–TIMS analyses at Princeton University

Mass spectrometry was performed using an IsotopX PhoeniX-62 TIMS. Lead analyses were performed using a two-sequence method in peak-hopping mode, switching the axial mass from 204 to 205. The axial mass was measured in a Daly photomultiplier ion counter and higher Pb masses were measured in Faraday cups. This allows for two static analyses in which mass 205 was used to monitor the Faraday-Daly gain in real time. A correction for mass-dependent Pb fractionation was applied based on repeated measurements of NBS 982 (Catanzaro *et al.* 1968) Pb [on both the Daly ion counter [$0.18 \times (1 \pm 0.02\ \%)$ amu^{-1}; 1*s*] and the Faraday cups [$0.09 \times (1 \pm 0.02\ \%)$ amu^{-1}; 1*s*] to account for differences in detector specific mass fractionation in the Daly setup. Deadtime on the Daly detector was also monitored by measuring NBS 982 over a large dynamic range up to a count rate of 2.5×10^6 s^{-1}. Although the calculated value drifts by about a nanosecond on a yearly time-scale, the correct value for the measurement period was applied to these data.

Uranium was measured as the oxide UO_2 by a two sequence method similar to Pb, but in this case alternating mass 272 and 267 in the Daly ion counter. The latter is used to monitor Daly-Faraday gain drift, and the former is used to monitor the $^{18}O/^{16}O$ composition used to correct for isobaric interferences on UO_2 that arise from, e.g., mass $^{18}O^{16}O^{233}U$ interfering with mass $^{16}O^{16}O^{235}U$. The Daly gain correction was applied to mass 272 ($^{18}O^{16}O^{238}U$). Uranium mass fractionation was monitored cycle-by-cycle from the deviation of measured $^{233}U/^{235}U$ from the known tracer $^{233}U/^{235}U$ using the EARTHTIME ET535 tracer composition reported in Condon *et al.* (2015).

Data reduction was performed using the programs Tripoli and U-Pb Redux (Bowring *et al.* 2011, McLean *et al.* 2011). All non-radiogenic Pb was attributed to laboratory blank with a mean isotopic composition determined by total procedural blank measurements. This uncertainty in this composition was negligible given the size of zircon fragments chosen for analysis.

References:

Bowring J.F., McLean N.M. and Bowring, S.A. (2011)
Engineering cyber infrastructure for U–Pb geochronology: Tripoli and U–Pb redux. Geochemistry Geophysics Geosystems, 12(6), Q0AA19.

Catanzaro E.J., Murphy T.J., Shields W.R. and Garner, E.L. (1968)
Absolute isotopic abundance ratios of common, equal-atom, and radiogenic lead isotopic standards. Journal of Research of the National Bureau of Standards – A. Physics and Chemistry, 72A(3), 261–267.

Condon D.J., McLean N., Schoene B., Bowring S., Parrish R. and Noble S.R. (2008)
Synthetic U–Pb 'standard' solutions for ID–TIMS geochronology. Geochimica et Cosmochimica Acta, 72(12S), A175–A175.

Condon D.J., McLean N., Noble S.R. and Bowring S.A. (2010)
Isotopic composition ($^{238}U/^{235}U$) of some commonly used uranium reference materials. Geochimica et Cosmochimica Acta, 74, 7127–7143.

Condon D.J., Schoene B., McLean N., Bowring S. and Parrish R. (2015)
Metrology and traceability of U–Pb isotope dilution geochronology (EARTHTIME tracer calibration part I). Geochimica et Cosmochimica Acta, 164, 464–480.

Corfu F. (2004)
U–Pb age, setting, and tectonic significance of the anorthosite–mangerite–charnockite–granite–suite, Lofoten–Vesterålen, Norway. Journal of Petrology, 45, 1799–1819.

Davydov V.I., Crowley J.L., Schmitz M.D. and Poletaev V.I. (2010)
High-precision U-Pb zircon age calibration of the global Carboniferous time scale and Milankovitch-band cyclicity in the Donets Basin, eastern Ukraine. Geochemistry Geophysics Geosystems, 11(1), Q0AA04.

Gerstenberger H. and Haase G. (1997)
A highly effective emitter substance for mass spectrometric Pb isotope ratio determinations. Chemical Geology, 136, 309–312.

Mattinson J.M. (2005)
Zircon U–Pb chemical abrasion ("CA–TIMS") method: Combined annealing and multi-step partial dissolution analysis for improved precision and accuracy of zircon ages. Chemical Geology, 220, 47–66.

McLean N.M., Bowring J.F. and Bowring S.A. (2011)
An algorithm for U–Pb isotope dilution data reduction and uncertainty propagation. Geochemistry Geophysics Geosystems, 12(6), Q0AA18.

McLean N.M., Condon D.J., Schoene B. and Bowring S.A. (2015)
Evaluating uncertainties in the calibration of isotopic reference materials and multi-element isotopic tracers (EARTHTIME tracer calibration part II). Geochimica et Cosmochimica Acta, 164, 481–501.

Schmitz M.D. and Davydov V.I. (2012)
Quantitative radiometric and biostratigraphic calibration of the global Pennsylvanian – Early Permian time scale. Geolical Society of America Bulletin, 124, 549–577.

Schmitz M.D. and Schoene B. (2007)
Derivation of isotope ratios, errors and error correlations for U-Pb geochronology using ^{205}Pb–^{235}U–(^{233}U)-spiked isotope dilution thermal ionization mass spectrometric data. Geochemistry Geophysics Geosystems, 8(8), Q08006.

Stacey J.S. and Kramers J. (1975)
Approximation of terrestrial lead isotope evolution by a two-stage model. Earth and Planetary Science Letters, 26, 207–221.
