# Supplementary material for: GZ7 and GZ8 – Two Zircon Reference Materials for SIMS U‐Pb Geochronology
Source: Geostand Geoanal Res. 2018 Oct 8;42(4):431–57. doi: 10.1111/ggr.12239 (PMC6334521; doi:10.1111/ggr.12239)
Supplement: Supplementary file 3 — Appendix S3. Documentation of locations of EPMA (Universität Göttingen, Germany) linescans, and plots and histograms of mass fractions of HfO2, ThO2 and UO2. [file GGR-42-431-s003.pdf]

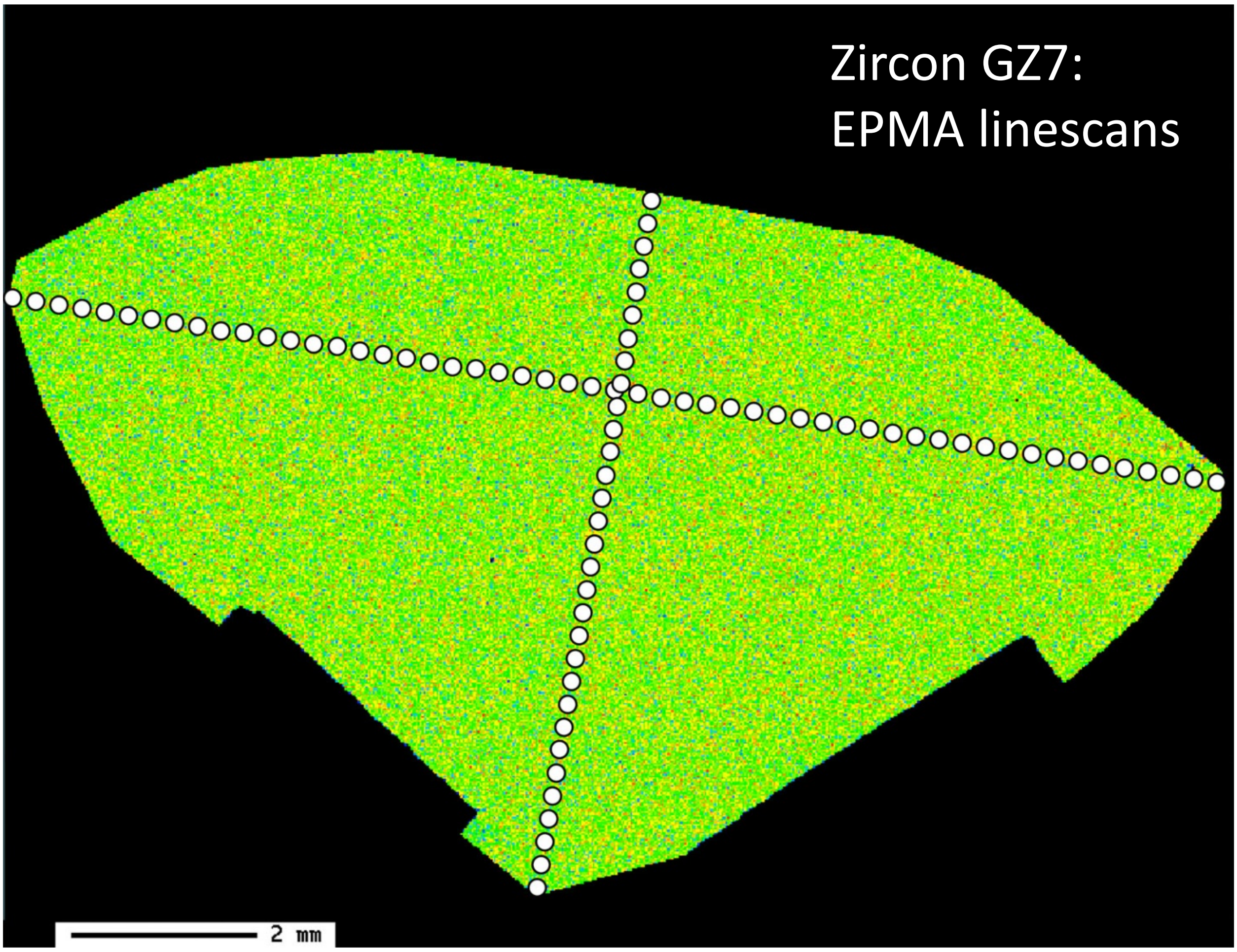
Zircon GZ7:
EPMA linescans
2 mm

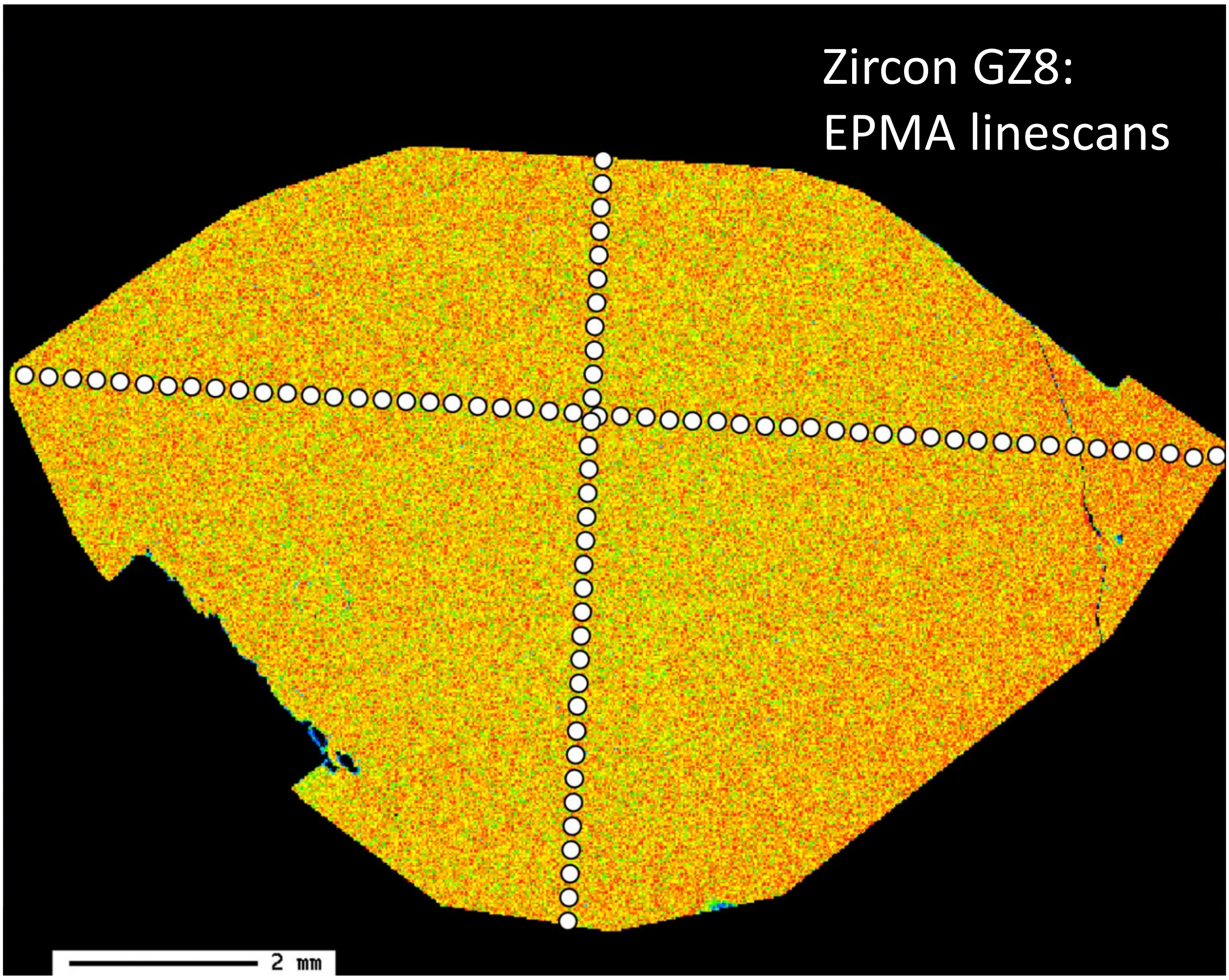
Zircon GZ8:
EPMA linescans
2 mm

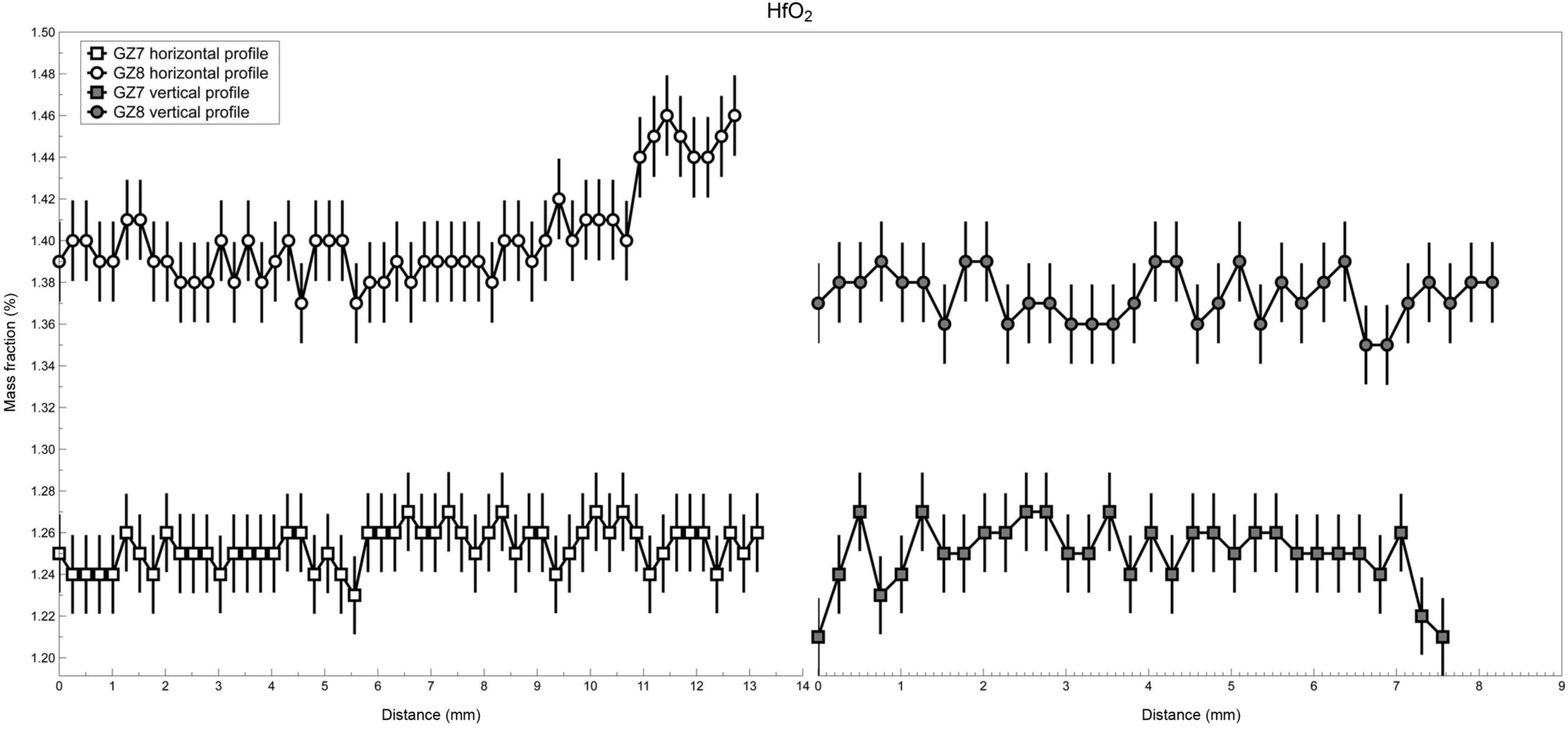

HfO_2
GZ7 horizontal profile
GZ8 horizontal profile
GZ7 vertical profile
GZ8 vertical profile
Mass fraction (%)
1.50
1.48
1.46
1.44
1.42
1.40
1.38
1.36
1.34
1.32
1.30
1.28
1.26
1.24
1.22
1.20
0 1 2 3 4 5 6 7 8 9 10 11 12 13 14
0 1 2 3 4 5 6 7 8 9
Distance (mm)
Distance (mm)

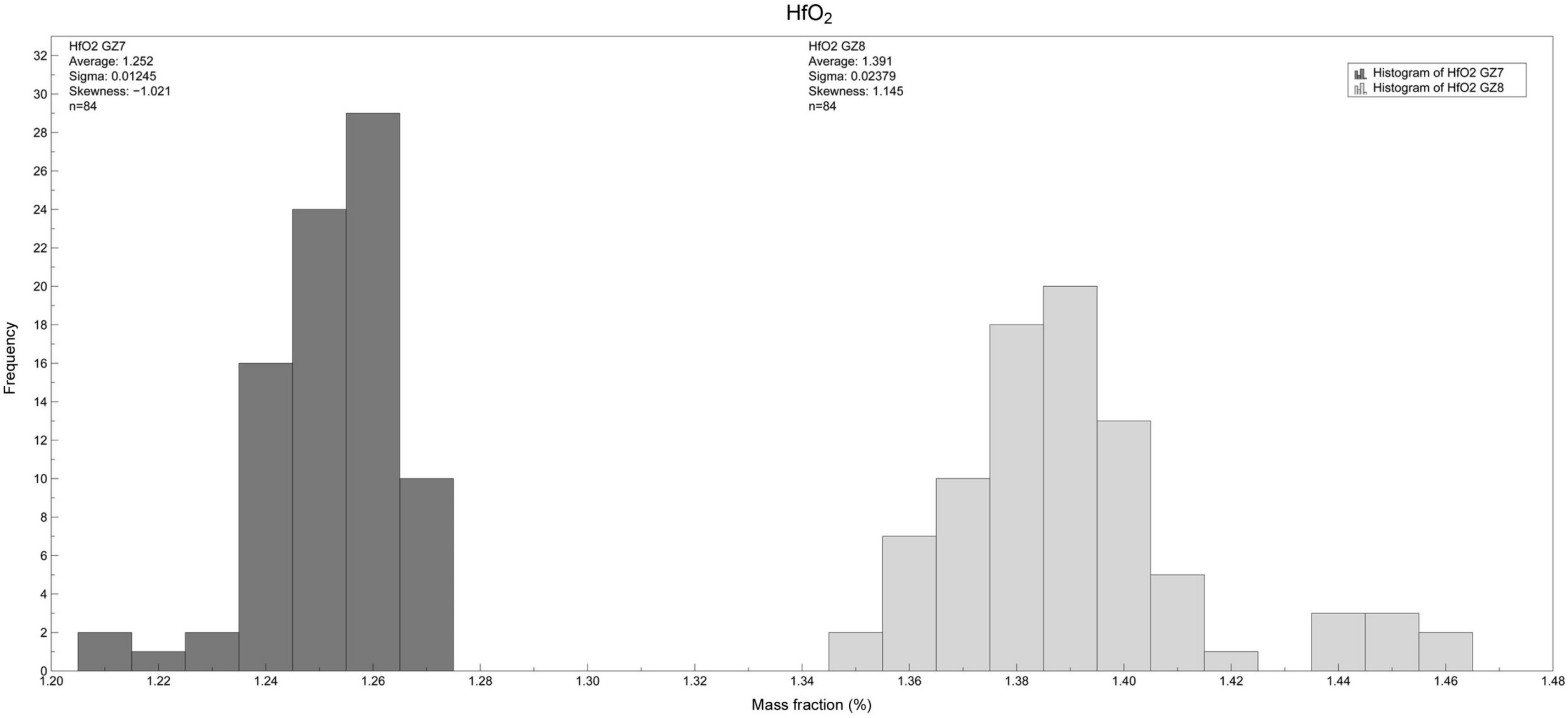
HfO2
HfO2 GZ7
Average: 1.252
Sigma: 0.01245
Skewness: −1.021
n=84
HfO2 GZ8
Average: 1.391
Sigma: 0.02379
Skewness: 1.145
n=84
Histogram of HfO2 GZ7
Histogram of HfO2 GZ8
Frequency
Mass fraction (%)
0
2
4
6
8
10
12
14
16
18
20
22
24
26
28
30
32
1.20
1.22
1.24
1.26
1.28
1.30
1.32
1.34
1.36
1.38
1.40
1.42
1.44
1.46
1.48

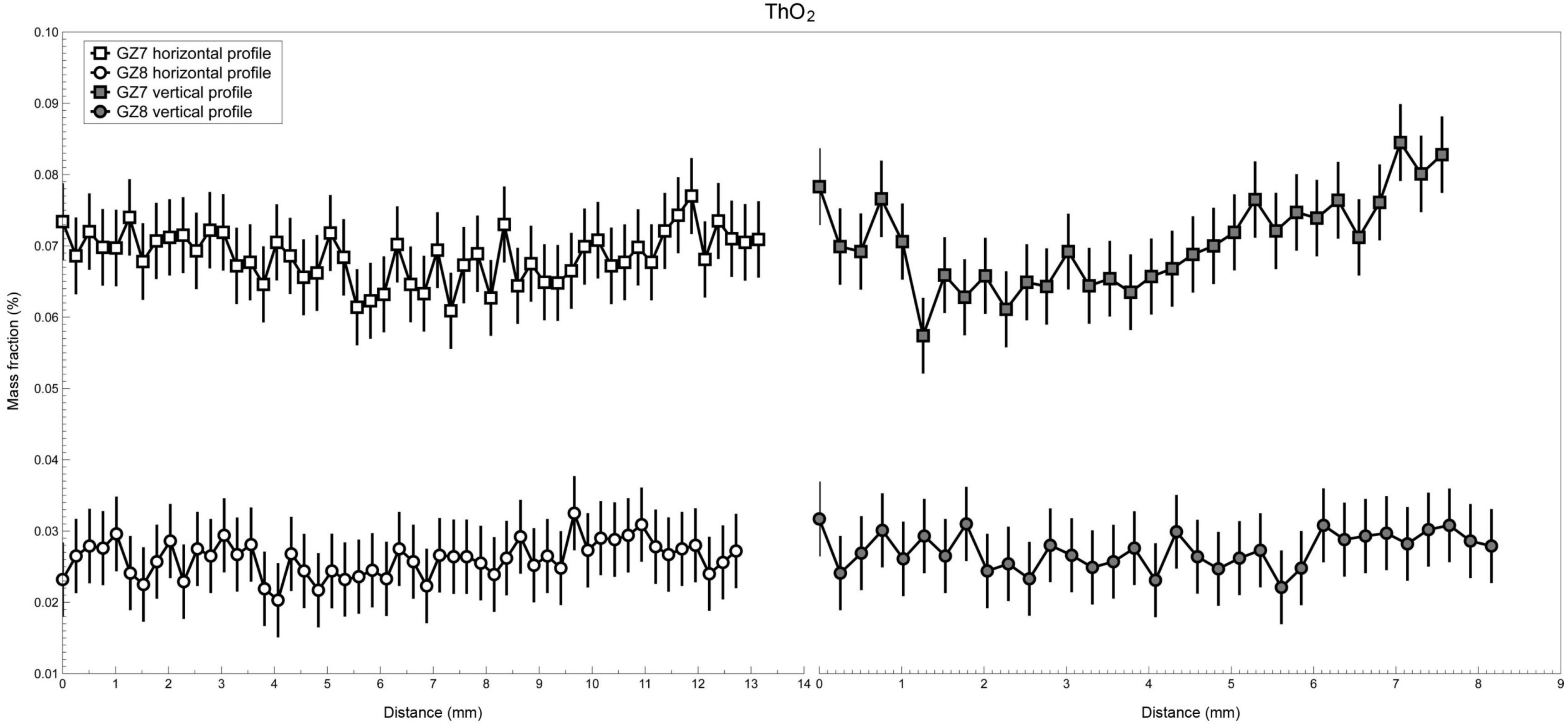
ThO2
GZ7 horizontal profile
GZ8 horizontal profile
GZ7 vertical profile
GZ8 vertical profile
Mass fraction (%)
Distance (mm)
Distance (mm)

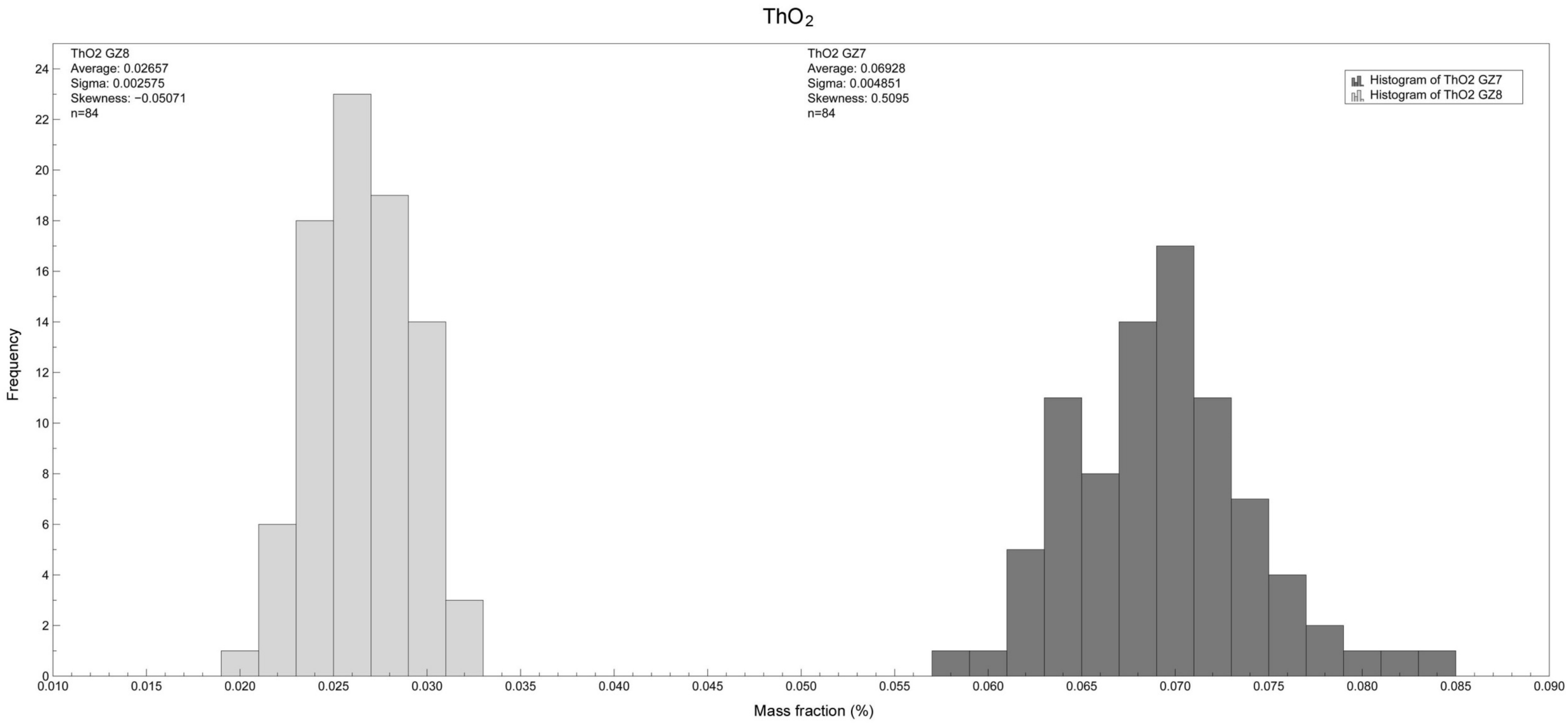

ThO2
ThO2 GZ8
Average: 0.02657
Sigma: 0.002575
Skewness: −0.05071
n=84
ThO2 GZ7
Average: 0.06928
Sigma: 0.004851
Skewness: 0.5095
n=84
Histogram of ThO2 GZ7
Histogram of ThO2 GZ8
Frequency
Mass fraction (%)

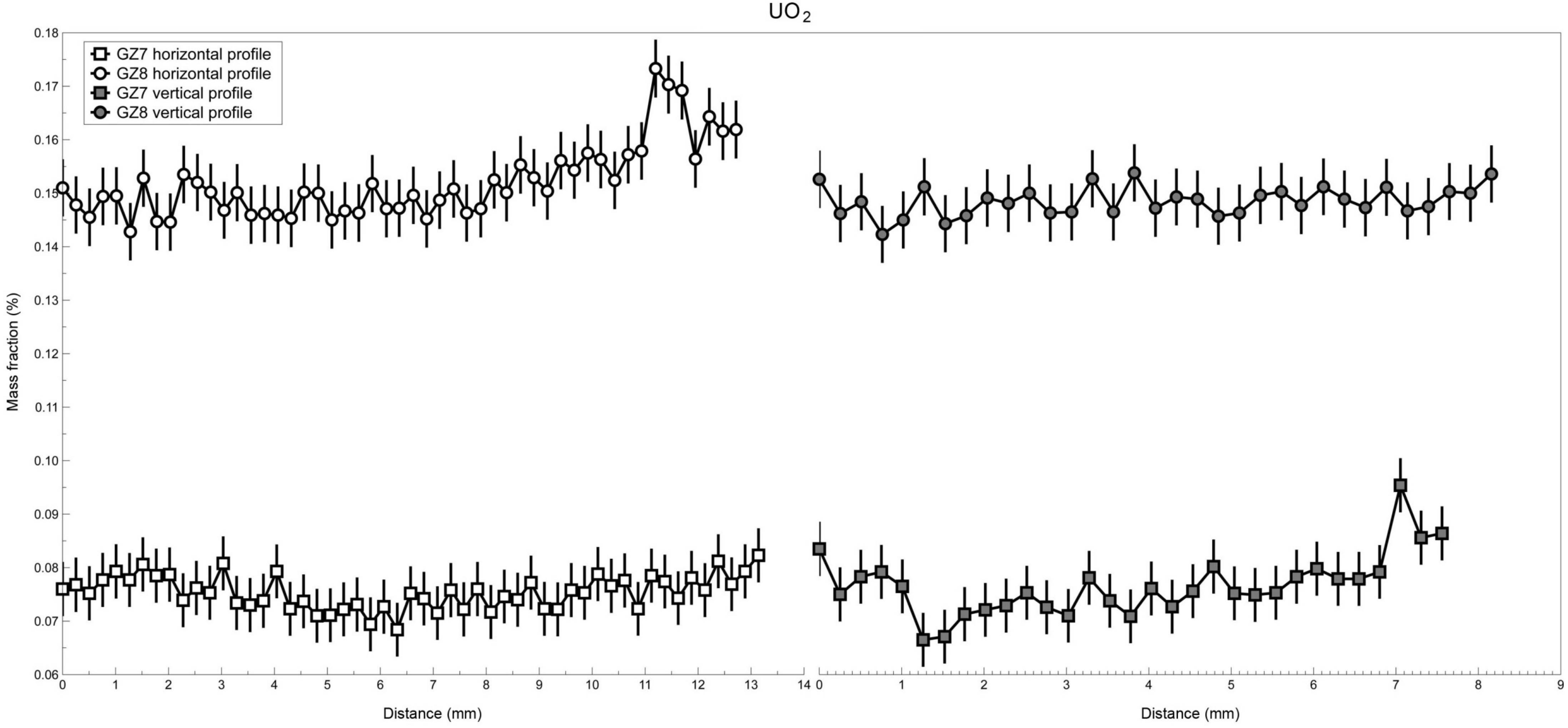

UO2
GZ7 horizontal profile
GZ8 horizontal profile
GZ7 vertical profile
GZ8 vertical profile
Mass fraction (%)
0.18
0.17
0.16
0.15
0.14
0.13
0.12
0.11
0.10
0.09
0.08
0.07
0.06
0 1 2 3 4 5 6 7 8 9 10 11 12 13 14
0 1 2 3 4 5 6 7 8 9
Distance (mm)
Distance (mm)

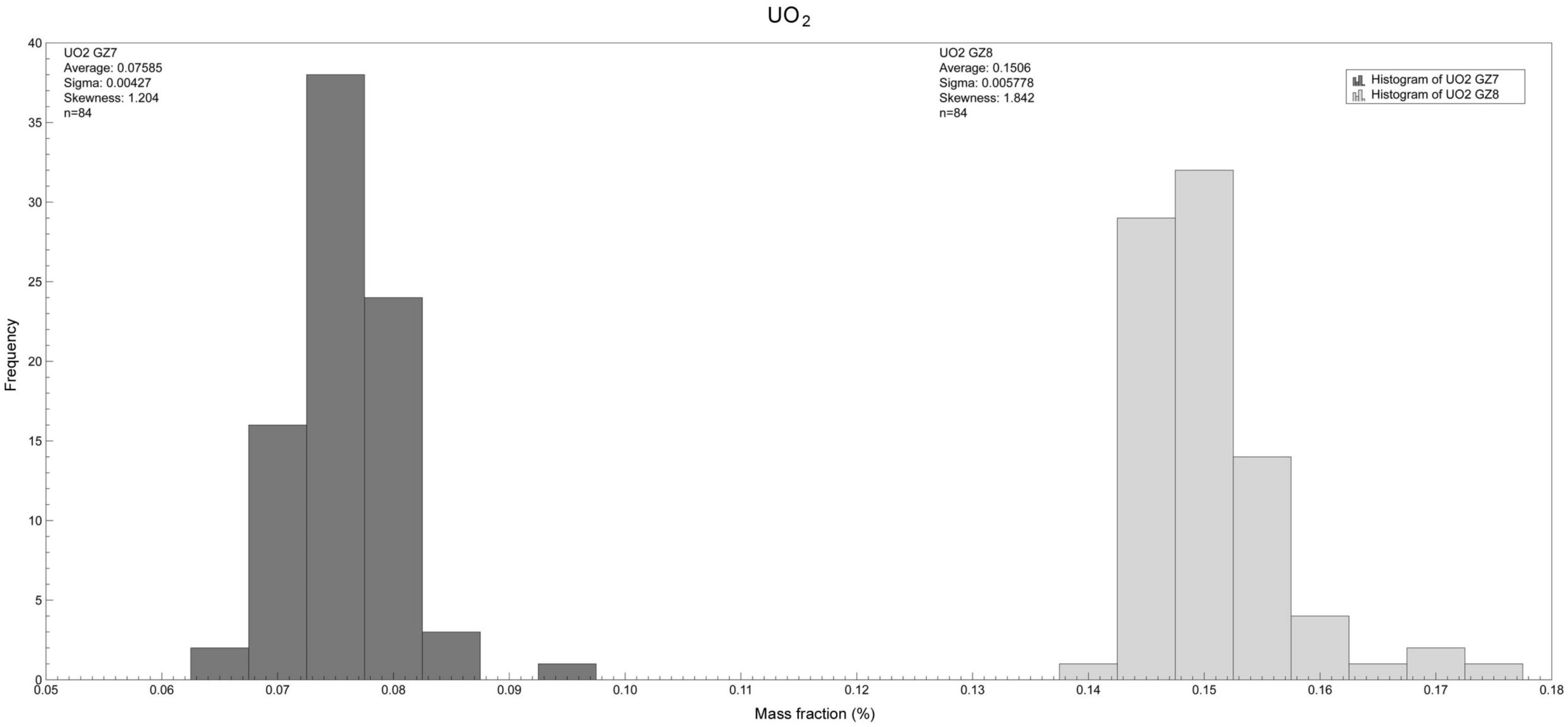
UO$_2$
UO2 GZ7
Average: 0.07585
Sigma: 0.00427
Skewness: 1.204
n=84
UO2 GZ8
Average: 0.1506
Sigma: 0.005778
Skewness: 1.842
n=84
Histogram of UO2 GZ7
Histogram of UO2 GZ8
Frequency
Mass fraction (%)
0 5 10 15 20 25 30 35 40
0.05 0.06 0.07 0.08 0.09 0.10 0.11 0.12 0.13 0.14 0.15 0.16 0.17 0.18
